# Agenda Setting and *The Emperor’s New Clothes*: People Diagnose Information Cascades During Sequential Testimony by Reasoning About Informants’ Speaking Order and Social Status

**DOI:** 10.1162/OPMI.a.258

**Published:** 2025-11-22

**Authors:** Emory Richardson, Isaac Davis, Frank Keil

**Affiliations:** Yale University

**Keywords:** sequential voting, informational independence, agenda-setting, collective behavior, consensus

## Abstract

Consensus-based social learning strategies often outcompete other strategies in evolutionary models. But while formal proofs suggest that consensus’ reliability is compromised when individual judgments are not independent, this makes for a notoriously implausible assumption in the biological world: the people we learn from are constantly learning from each other as well. How do we avoid being misled by consensus? We present three experiments and a computational model examining commonsense reasoning about how people’s public and private judgments are influenced by the consensus and social status of those around them. Results suggest that while people realize that these two factors can cause others’ public and private judgments to diverge, their own trust in public consensus depends on how accurately they believe it reflects their informants’ true beliefs.

## INTRODUCTION

Imagine yourself on a five-person engineering team needing to decide which of two model airplane designs would fly the best in a contest. Each teammate first evaluates the airplanes privately, and then, one-by-one, announces their vote. This means that only the first speaker votes without knowing anyone else’s vote, and knowing earlier speakers’ votes could change later speakers’ public votes—and their private beliefs—for better or for worse. But it may not affect them each in the same way, and might not affect them at all. As a truth-seeking fifth speaker, you’d like to use the design that’s actually best, and other people’s judgments might help you decide between them—but how can you distinguish the signal from the noise?

The inference problem you face is trickier than it is for earlier speakers: in order to weigh your own judgment against the previous speakers’, you’ll also need to evaluate speaker one’s influence on two, their joint influence on speaker three, and so on. But it’s not a problem easily dismissed as an artifact of an unusual voting process: many social contexts involve some form of sequential belief updating at micro- and macro-scales, from turn-taking in conversation and parliamentary voting to viral tweets and the influence of yesterday’s trades on today’s stock prices. And it’s a problem with teeth: your decision to trust or distrust that chain of influences not only affects you, but the downstream observers influenced by your decision, who may later influence you in turn. Socially transmitted information can be useful—cumulative culture would be impossible without it—but it can also lead to catastrophic information cascades (Boyd & Richerson, 1995; Raafat et al., 2009), where what appears to be a large consensus of independent opinions is actually the result of a small number of opinions propagating through the network. So how do we learn from social information without being misled by it? Here, we focus on commonsense reasoning about how public and private judgments are influenced by *social favor* (the desire to align oneself with or against an individual or group) as well as *information cascades* (herd behavior by rational agents exposed to sequential decision-making processes). We examine this reasoning using human data from three experiments and a computational model that allows us to investigate the intuitions that drive human judgments in closer detail.

Here’s the gist of our argument. Large literatures demonstrate that both high-status (i.e., dominant, prestigious) individuals and strong consensus can put considerable pressure on people’s public and private judgments (Jiménez & Mesoudi, 2019; Kameda et al., 2022; Morgan et al., 2012; Pink et al., 2021; Raafat et al., 2009). In some cases, this may be because consensus and status are heuristics for accuracy. In other cases, people may simply want to align themselves with an individual or group—a desire for social favor, even against their better judgment. But these pressures aren’t abstruse discoveries of 20th century academic psychology. They’re part of the commonsense psychology adults use to both interpret and influence each others’ behavior (Mahr & Csibra, 2022; Ullman & Tenenbaum, 2020). Our suggestion is that commonsense reasoning about how they play out in sequential updating processes can make people more vigilant about the potential for information cascades. But existing evidence of people’s vigilance in sequential updating scenarios is mixed (Anderson & Holt, 1997; Fränken et al., 2024; Whalen et al., 2018; Xie & Hayes, 2022). We think that this is not because people are insensitive to the potential for information cascades. Instead, we’ll argue below that understanding people’s reliance on socially-transmitted information requires looking closely at how they expect it to affect *other* people’s beliefs. If one informant in a sequence believes more or less everything they hear, another trusts nothing but their own eyes, and a third will publicly endorse whatever plays to their advantage even if it contradicts their private beliefs, what an observer can learn from them can just as easily depend more on the informants’ speaking order than on the informants’ ability to evaluate evidence for themselves, *or vice-versa*. We think people know this, and it influences the way they reason about sequential testimony in lab experiments.

### (In)sensitivity to Information Cascades in Sequential-updating

Learning from Bob lets Alice avoid the costs of learning alone; but she also risks inheriting his errors. And the errors may not even be his. Bob may have learned from Carol, who learned from David, and so on. In other words, social learning allows *errors* to cascade through long transmission chains as well as knowledge. In some contexts (e.g., hearsay), the risks of relying on second- and third-hand information seem intuitively obvious (Altay et al., 2020). But less intuitively, a few relatively mild assumptions make deferring rational in some contexts. Namely: if Alice assumes Bob’s judgment reflects the evidence available to him, then even a relatively small consensus may provide her with sufficient evidence-of-evidence to override *whatever* her private evidence suggests. And evidence of evidence *is* evidence (Hedden & Dorst, 2022; Feldman, 2014). For instance, suppose that given a binary choice, Alice’s private evidence says that Bobcat Bite makes a better burger than Louis’ Lunch. But, she sees that Bob and Carol have chosen Louis’ Lunch; if Alice assumes that these decisions reflect their (also binary) evidence, then her evidence consists of two votes for Louis’ (Bob and Carol) and one for Bobcat (her). Being a rational agent, she changes her mind and goes to Louis’—leaving the next voter facing an even stronger consensus. In other words, rational deference allows a relatively small number of early votes to cascade through a chain. And since humans (like many species) treat consensus strength as evidence of decision quality (Morgan et al., 2012), a cascade might quickly gather enough momentum to survive the occasional dissenter.

The paradoxical implication noted in two seminal papers (Banerjee, 1992; Bikhchandani et al., 1992) is that as soon as consensus is strong enough to make your informants conform regardless of their private evidence, it’s also strong enough to undermine the assumption that public consensus reflects their private evidence. Don’t mistake our point, though: if the goal is *accuracy*, the load-bearing assumption is *not* that public consensus reflects private evidence. In some cases, deferring to the first few voters might improve accuracy (e.g., if they’re the only ones with any relevant expertise, or if their votes are akin to a lookout’s emergency alert and false alarms are comparatively low-cost). The load-bearing assumption, formalized in models originating with Condorcet and Galton, is that informants’ *errors are statistically independent*—because if *errors* are independent, the only bias left that can consistently produce consensus is the informants’ *knowledge*. So the problem with sequential voting is that since it undermines the assumption that informants’ judgments are independent, it also leaves them guessing whether consensus is evidence of the strength of the informants’ discernment as individuals, or the strength of their deference to the first few votes. Strong discernment makes consensus a reliable cue to accuracy; strong deference could make it either more reliable than that or less, depending whether the early speakers are more or less reliable than the people deferring to them. The mere existence of consensus doesn’t tell us which it is; but some theory-of-mind reasoning might.

Given the importance of information cascades to theories of biological and cultural evolution, one might expect learners to be sensitive to their risks as much as their benefits. But evidence from the several existing studies is mixed (Anderson & Holt, 1997; Whalen et al., 2018; Xie & Hayes, 2022). Participants in these studies are presented with decisions akin to Alice’s Bobcat Bite scenario. An urn is filled with colored balls in an 80:20 proportion favoring either red or blue. One urn is randomly selected, and after privately sampling a ball from the urn, each informant announces their inference about which color they believe predominates—either publicly to the participant and the remaining informants (potentially influencing those informants’ judgments), or privately to the participant alone (ensuring that the informants’ judgments are independent of each other). But when participants in these studies saw every informant infer the same color, they were just as willing to defer to the unanimous consensus regardless of whether it was public or private (Xie & Hayes, 2022). Moreover, even when experimenters showed participants both sequences and asked them to explain whether or not one would be more informative than the other, participants defended each in approximately equal proportions (~35–40%, with 25% indifferent). These findings are consistent with earlier work (Whalen et al., 2018) in which participants’ skepticism was only piqued if (A) one of three informants dissented or (B) all three informants jointly sampled a single ball to make their judgments instead of each sampling their own.

Why aren’t people more skeptical of consensus when their informants might have influenced each other? The kind of statistical independence demanded of consensus by Condorcet and Galton is certainly appropriate to guesses about randomly selected urns—especially considering that using 80:20 proportions means that adult human informants are unlikely to come anywhere near falling below the minimum individual competence threshold that makes consensus reliably accurate instead of reliably *in*accurate. But like others (Dietrich & Spiekermann, 2013; Laan et al., 2017; Whalen et al., 2018; Xie & Hayes, 2022), we think it’s noteworthy that widespread social learning makes that degree of statistical independence essentially non-existent in the real world. And in real-world social learning, allowing informants to influence each other—even directly—can make consensus *more* reliable instead of less (Barnett, 2019; Kao et al., 2014; Mericer & Claidière, 2022; Pilditch et al., 2020; Pfänder et al., 2025; Toyokawa et al., 2019; Trouche et al., 2014). So real-world social learners can’t disregard consensus simply because informants *might* have influenced each other. They have to decide whether any of their informants *were* influenced, why, and whether that would make consensus more reliable instead of less. But both learners and their informants often rely more heavily on their *own* judgment relative to other people’s than models imply is optimal (Mannes, 2009), and when they do defer to each other or consensus, they’re selective about *whose* judgment they trust, how much, and why (Harris et al., 2018; Kameda et al., 2022; Morgan et al, 2012). And unlike participants’ real-world informants, the informants in the balls-and-urns task are identical in every sense but their speaking order. So, prior to the vote, participants’ real world experience would make them unlikely to suspect that their informants would weigh the previous speaker’s guess (e.g., “mostly blue”) more heavily than their own evidence (e.g., a red ball). And in contrast to the impact of social learning on real-world consensus, the balls-and-urns task is designed to make it impossible for consensus to be more reliable if any informant defers to the previous speaker’s guess. But the only way for participants to see this is to both (1) accurately compute how hearing those guesses changes the conditional probabilities available to each successive informant and (2) assume that all the informants *would* make the Bayes-normative move of weighing the previous informant’s guess more heavily than their own evidence. In short, the kinds of signals the balls-and-urns task provides may simply not trigger strong vigilance to the risks of information cascades.

### Diagnosing Pluralistic Ignorance

Richer contexts may elicit stronger skepticism. For instance, people are mindful of alliance-based biases in testimony. If Jack endorses Jill, his endorsement seems less informative if Jack and Jill are close friends than if they dislike each other; and vice-versa if Jack disparages her. Even children make this inference (Liberman & Shaw, 2020), and by adulthood we use it to reason about the evidential value of consensus: if one of the three eyewitnesses testifying about Jill’s alibi as a suspect in a robbery is her friend Jack, his testimony only counts towards consensus if he contradicts her alibi, not if he endorses it (Mercier & Miton, 2019). You may not even expect Jack believe his own testimony; it simply reflects his desire to maintain his relationship with Jill. A similar kind of reasoning may help explain why people so easily dismiss the opinions of millions of political opponents (Oktar & Lombrozo, 2024): Jack may seem no less well-informed and genuine in his beliefs than you are, but if he holds an opinion that only became widespread after being espoused by party leaders, dismissing it as dogma is easy.

The Jack and Jill examples illustrate two basic intuitions that constrain our reasoning about socially transmitted beliefs. First, we think that Jack will have to weigh his desire to align himself with Jill alongside a variety of other values, both epistemic and non-epistemic; sometimes that calculus will reinforce his private beliefs or generate entirely new ones, but other times it may cause him to take public positions that conflict with what he privately believes. In other words, reasoning about how much Jack cares about accuracy relative to social favor can help us estimate the evidential value of Jack’s testimony. Second, we think that the influence Jack’s relationship with Jill has on that calculus depends on what he knows of Jill’s stance—i.e., what she herself thinks and how she’ll respond to him taking one position or another. For instance, put Jack and Jill in the engineering team scenario. The only way to be sure that Jack’s vote reflects his private beliefs more than his desire to align himself with Jill is if he speaks before she does. But suppose Jill speaks first and Jack speaks second; if a rational third voter like Alice is unaware that Jack was just currying favor, she might trigger an information cascade by deferring to their two votes, even if social favor plays no role in her own judgment at all. Or suppose that Jill speaks first and Jack speaks last: if Jill voted for one design and everyone else voted for another, Jack not only has to weigh Jill’s social favor against the rest of the team’s and any evidential value of Jill’s prestige against the evidential value of a 3-to-1 consensus—he also has to keep in mind that since groups often default to majority rule (Boehm, 1996; Laughlin, 2011), his vote may not affect the outcome anyway.

In short, reasoning about social favor makes it intuitive that earlier speakers not only have more influence over later speakers than vice versa; it also suggests that even with a private consensus strongly against them, someone with disproportionate power may be able to swamp later speakers’ desire for accuracy simply by speaking first—at least under certain conditions (Levine & Plott, 1977). The difficulty of contradicting a dominant or prestigious first speaker (or an emerging consensus) might make information cascades more likely, especially for someone who “naively” expects their informants to always-and-only vote their beliefs. But intuitions that allow people to titrate epistemic and social influences on their informants’ public and private judgments might make people better at detecting information cascades before they start—and make them less likely as a result. If so, then understanding people’s reliance on socially-transmitted information will require looking closely at both the epistemic and social motivations they believe *others* have for accepting or rejecting it. Our goal in this paper is to examine the relationship between people’s trust in sequential consensus and their inferences about their informants’ private and public judgments. Broadly, our pre-registered prediction^*^ is that we can make participants less trusting of a sequential consensus by increasing the first speaker’s prestige (but not by increasing the last speaker’s prestige), because a prestigious first speaker will lead them to expect greater gaps between the subsequent speakers’ public and private judgments, but a prestigious last speaker won’t. We also pre-register a computational model that parameterizes participants inferences’ about the epistemic and social influences on their informants’ public and private judgments.

## GENERAL METHODS

**Figure 1.**
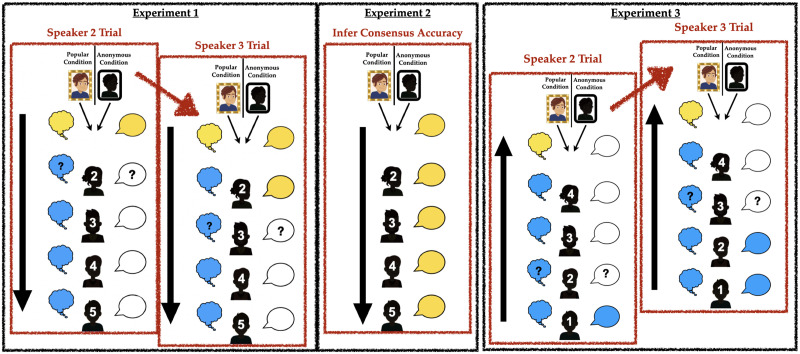
Procedure and example stimuli for Experiments 1–3. In Experiments 1 & 3, thought bubbles depict each agent’s private beliefs, while speech bubbles depict public votes. Participants see all agents’ private beliefs & the first agent’s public vote. They then predict each subsequent agent’s public vote and private belief, iterating through remaining agents. In Exp 1, the focal agent speaks first; in Exp 3, they speak last. In Exp 2, participants only see agents’ public votes and rate their own trust in the public consensus.

We use the engineering-team scenario introduced earlier to ask people to reason about how much influence early speakers’ public “votes” have on subsequent speakers’ public votes *and* their private beliefs. Participants are told that initially, four teammates privately believe the blue airplane design is best, and one privately believes the yellow design is best (Figure 1). We then tell them that the contest organizers asked the teammates to announce their votes by going around from left-to-right, and reveal that the first speaker votes their beliefs. Critically, we manipulate participants’ perceptions about the speakers’ potential social motivations in two ways: first, by introducing the Yellow teammate as either “very popular” or omitting mention of their social status, and second, by making them either the first speaker (in Experiments 1 and 2, thus starting the sequence with a vote from the single informant who privately dissents) or last speaker (in Experiment 3, thus starting the sequence with a vote from one of the informants in the private majority).

In Experiment 1, participants first rate which design the second speaker is relatively more likely to vote for publicly after hearing the first speaker’s vote, *and* which design the second speaker is relatively more confident in privately. We then ask participants to make the same inferences for each subsequent speaker, assuming their judgment about the previous speaker was correct. For instance, if they expect speaker two to vote yellow, we ask them what speaker three will publicly vote and privately believe after hearing speakers one and two vote yellow. Experiment 2 is similar, but participants are not shown the speakers’ initial beliefs. Instead, we show them that following the first speaker’s yellow vote, each subsequent speaker also voted yellow. Finally, Experiment 3 is identical to Experiment 1 except that the speaking order is reversed—meaning that participants see the first speaker vote blue, and are then asked to infer the votes and beliefs of each subsequent speaker, including the Yellow teammate. This allows us to ask whether participants expect speakers’ influence over each other to depend on speaking order as much as status, as well whether information cascades have as much influence on high-status speakers as anyone else. At the end of each experiment, participants are also asked which airplane they themselves believed was best, and which they believed the team would choose. The accuracy question provides the most direct comparison to the traditional balls-and-urn task as a measure of the sequence’s influence on participants, particularly in Experiment 2; the team choice question allows us to probe intuitions about whether or not sequential belief updating can create a consensus durable enough to survive when people have an opportunity to share second thoughts in back-and-forth deliberation. On the one hand, open discussion might make a conflict between public and private consensus easier to discover (Mercier & Claidière, 2022; Richardson & Keil, 2022; Trouche et al., 2014); on the other hand, it could also give a persuasive (or powerful) dissenter more opportunities to influence other informants.

Our experimental design differs from the original balls-and-urn task in several key ways. The design of the balls-and-urn task strongly implies that a speaker’s *pre-sequence* beliefs will only be inaccurate as a result of bad luck, rather than than bad judgment. That’s because with opposite 80:20 proportions—which would be unmistakable for any adult human—informants will be mutually aware that each randomly sampled ball is highly likely to be diagnostic of the predominant color, and the only relevant evidence that any of them can have. This is important for two reasons.

First, it conspicuously minimizes the social pressures that can arise from public disagreement, especially among the first two or three speakers: if the second speaker contradicts the first, the only implication is that one of the two happened to draw the minority ball, not that one of them wasn’t competent enough to understand the evidence. In that context, participants wouldn’t expect the second speaker to agree with the first speaker unless their ball colors matched. And second, it makes the odds of *starting* a sequence with two misleading balls minimal (4% of sequences, vs 64% starting with two truth-conducive balls, and 32% with split evidence). Taken together with the implication that the second speaker had no reason to flip their vote, *not* getting a split vote from the first two speakers is strong evidence that the first two balls *were* diagnostic.

Our point here is not that informational conformity akin to the Bobcat Bite scenario we described earlier couldn’t play a role in speakers’ judgments (particularly later speakers’ judgments)—just that participants wouldn’t expect it to be enough to flip the second speaker’s vote. Put more broadly, a random sampling task with such unmistakable ground truth proportions minimizes social pressure in a way that would make participants less vigilant to information cascades. Since our claim is that people’s vigilance about social influences may be a catalyst for detecting information cascades, we modified the balls-and-urn task in several ways.

First, instead having each informant observe separate evidence randomly sampled from urns, the informants in our task all observe the same underlying evidence, by privately assessing the two engineering designs. This makes social pressure more salient: if speaker 2 contradicts speaker 1, it *does* imply that one of their beliefs is a result of bad judgment, rather than bad luck—which we think is more representative of real world inference. Second, instead of setting ground truth by stipulating the proportions in each urn, we reveal the informants’ pre-sequence beliefs to participants—and instead of revealing the next informant’s vote at each round, we ask participants to *predict* the next informant’s vote *and* belief at each round. These latter two changes mean we have to forfeit direct comparisons to the balls-and-urn task. But, they allow us to more directly address the question we aim to study—the relationship between people’s reliance on socially-transmitted information and their beliefs about the epistemic and social motivations they believe *their sources* had for accepting or rejecting it.

With those two caveats in mind, we did attempt to align our task with the balls-and-urn task to the extent possible. First, we deliberately chose a 4:1 pre-sequence consensus, because that’s the ratio of evidence most likely to occur in a balls-and-urn task using 80:20 proportions other than a unanimous sample (which makes worries about information cascades moot). And we deliberately arranged it to *start* with the minority judgment because the concern that motivates the balls-and-urn task is that, since sequential voting amplifies early votes, misleading evidence from the first voter has the most power to produce a “false consensus”. In short, we think our task sheds light on the same reasoning processes being studied in the balls-and-urn task, but highlights the potential for social pressure that the original design obscured.

### Computational Framework

To better understand participants’ intuitions in this task, we posit a parameterized, generative model of how participants expect informants to update their beliefs and decide how to vote. By fitting the parameters of the model to participant judgments, we obtain a clearer picture of how participants’ expectations about the informants drive their predictions. At a high level, our model assumes that each informant decides how to vote publicly by weighing their private beliefs about which option is better against their desire to gain social favor—which they do by publicly agreeing with one set of informants or another. This means that their public and private judgments may diverge if their desire for social favor outweighs their desire for accuracy. However, social favor doesn’t influence any informant’s private beliefs directly; it can only influence them if it drove *previous* speakers to vote contrary to their own beliefs.

Here’s how it works. Suppose that after privately investigating both kits, each informant forms an initial private belief about which one is likely better. For the *n*th informant, we denote this initial private belief as a pair of probabilities [Y_n_, B_n_] that each option (yellow or blue) is better. Note that, unlike in the ball-an-urn task, we are deliberately agnostic about what what inference process led them to their initial private belief, and simply take these initial beliefs as given. This allows participants to focus on how informants will weigh their private evaluations against the opinions and favor of others, without having to track or compute any objective probabilities. Drawing on prior work (Toyokawa et al., 2019; Whalen et al., 2018; Xie & Hayes, 2022), our model assumes that as more informants make their votes public, subsequent informants will treat those public votes as a kind of indirect evidence (“evidence of evidence”), and will update their private beliefs accordingly. The rate at which each informant updates their beliefs depends on two factors. First: how confident they are in their own private opinion, relative to the opinions of others, which we denote by W_Self_. Intuitively, we can think of this as the number of “votes” each informant assigns their own private opinion relative to the opinions of others. Second: whether and to what degree they treat numerically stronger majorities as a proxy for stronger evidence, which we denote by an exponent *θ*_infoCon_ (“informational conformity”). Given these parameters, and the number of V^Y^_-n_ of prior informants who voted for Y, we define the nth informant’s updated degree of belief in option Y as:

$${\text{belief}}_{n}^{Y}=\frac{{W_{n}}^{\text{self}}\cdot Y_{n}+{\left(.01+{V^{Y}}_{-n}\right)}^{\theta_{d}}}{{W_{n}}^{\text{self}}\cdot Y_{n}+{\left(.01+{V^{Y}}_{-n}\right)}^{\theta_{d}}+{W_{n}}^{\text{self}}\cdot B_{n}+{\left(.01+{V^{B}}_{-n}\right)}^{\theta_{d}}}$$

Where our model diverges from prior work, however, is our assumption that in addition to tracking how informants update their beliefs in each option, participants may also track the amount of *social favor* associated with each option. That is, in a social context where informants may have other, non-epistemic motivations for voting, one potential motivation is the desire to gain social favor by publicly agreeing with one’s peers: the more social favor associated with each option, the greater the potential for socially-motivated voting. To this end, we define a *social power* parameter W_Power_ which represents the nth agent’s social status within the voting group. This allows us to represent a scenario in which some informants (e.g.: the “popular” informant) carry more social power with the group than others, making their votes “count more” when computing the total social power associated with each option. Formally, let P^Y^_−n_ be the total social power associated with option Y, which we compute as ∑_i < n_ **I**[v_i_ = Y]* W_Power_ (i.e.: the number of votes for Y, weighing each vote by that informant’s social power). We define the total social favor associated with option Y after n−1 votes as:

$$\text{inf}{{\text{luence}}^{Y}}_{n}=\frac{{\left(.01+{P^{Y}}_{-n}\right)}^{\theta_{s}}}{{\left(.01+{P^{Y}}_{-n}\right)}^{\theta_{s}}+{\left(.01+{P^{B}}_{-n}\right)}^{\theta_{s}}}$$

where *θ*_socCon_ is a *social conformity* parameter analogous to *θ*_infoCon_. Finally, given the updated degree of belief in option Y, and the amount of social favor associated with option Y, we assume that the probability of the nth informant voting for Y is a weighted sum of these two terms, where the weighting parameter *ω*_acc_ reflects how that informant values accuracy (i.e.: voting for what they think is correct) versus social influence (i.e.: voting to gain social favor within the group). A *ω*_acc_ value near 1 represents an informant who almost always votes in line with what they actually believe, regardless of which option is more “popular” (i.e.: carries more social favor). Conversely, a *ω*_acc_ value near 0 represents an informant who disregards their own private beliefs and simply votes for whichever option carries more social favor. Formally, we define informant n’s probability of voting for Y as

$$P\left(v_{n}=Y|V_{-n}\right)=\omega_{acc}\cdot {{\text{belief}}^{Y}}_{n}+\left(1-\omega_{acc}\right)\cdot \text{inf}{{\text{luence}}^{Y}}_{n}$$

Thus, equations 1–3 define the generative model which we posit to capture participant intuitions as they predict the private beliefs and public votes of the informants. By fitting the model parameters to each participants’ judgments, we can estimate each participant’s beliefs about the informants’ use of social information, and predict how these beliefs drive participant predictions about each informant’s public and private judgments. Furthermore, in order to isolate whether participants are, in fact, sensitive to the potential effects of disproportionate social power in a sequential voting scenario, we compare our full model (varPower) as defined above to a lesioned model, (fixedPower) in which WPower is set to 1 for all informants. Intuitively, the fixedPower model represents an observer who expects all informants to have equal social status, while the varPower model allows some informants to have more or less social status than others. If participants are, in fact, reasoning about the agents’ disproportionate social power throughout the task, we should find that the optimized varPower model outperforms the optimized fixedPower model in tracking participant judgments *only* when the dissenting speaker a) is identified as “popular” and b) speaks first in the sequence. In addition to testing these models against human data, the supplemental materials includes an analysis of the effect of these parameter combinations by simulating each speaker’s predicted vote independently at each round (e.g., even if a given parameter set made the probability of a Y vote from Spkr2 negligible, we still simulated Spkr3’s vote under both a YY and a YB pattern).

## EXPERIMENT 1

In Experiment 1, we presented mTurkers with the scenario outlined in the General Method (color-coding the airplanes yellow or blue, to prevent participants from seeing the designs for themselves). We manipulated social favor by either describing the first speaker as “*very popular”* or omitting mention of status. As outlined in our computational model above, we predicted that as compared to participants in the Anonymous condition, participants in the Popular condition would rate subsequent speakers’ private beliefs and public votes as shifting further towards yellow from blue, and rate them as more likely to “flip” their public votes to yellow despite still privately believing blue.

### Participants

Based on piloting, we aimed for a final sample of 120 respondents per condition (Popular or Anonymous) from mTurk. Respondents were required to complete basic comprehension checks about the instructions in order to advance to the survey itself. After screening out *n* = 40 mTurkers prior to participating for failing these checks and *n* = 3 for botlike explanations (per our pre-registration), our final sample included *n* = 120 in the Anonymous condition and *n* = 118 in the Popular condition.

### Procedure

Participants were introduced to a five-person team building a remote control airplane for a contest using one of two designs. The designs were presented in color-coded (yellow or blue) boxes in order to prevent participants from evaluating them directly. They were told that the contest organizers asked each person to look in each box and think silently about which would be best for the contest. A thought-bubble next to each person showed their private belief: one person privately believed the yellow plane was best, while the remaining four all believed the blue plane was best. Next, participants were told that the contest organizers asked the teammates to go around one-by-one from left to right and say which kind of airplane they thought was best. Then, they were told that (given the left-to-right order), the first person to speak was the teammate who privately believed yellow, and that they voted yellow. In the Anonymous condition, the teammate was left unnamed; but in the Popular condition, he was introduced as “Max”, and described as being very popular.

We asked participants to infer (1) what the second speaker would *say* and (2) what they privately *believed*, reminding them that while the teammates had heard the first speaker (aka Max) vote yellow, none of them knew what the subsequent speakers would vote or what they believed. For each judgment (*believe* and *say*), participants responded using a 20 point scale, ranging from 1 (definitely [believes/will vote for] blue) to 20 (definitely [believes/will vote for] yellow). By having participants explicitly rate the probability of each agent believing/voting for each option (as opposed to a binary prediction), we can directly compare participants’ predictions to the voting and belief probabilities predicted by our model. On each subsequent trial, they were asked to assume their answers for the previous round were correct For instance, if a participant predicted that the second speaker would vote for yellow with probability > .5, then on the next round that same participant would be asked what the third speaker would privately believe and publicly say after hearing both Max and the second speaker say yellow.^1^ Finally, we said that after voting, the contest organizers asked the team to talk together to make a final decision, and asked participants to infer (1) which design the team would settle on, and (2) which design they themselves thought was best. Participants responded to all questions using a 20-point scale anchored at 1-“definitely blue” and 20-“definitely yellow”; for the final-team-decision and true-best questions, they were also asked to briefly explain their answers.

### Results and Discussion

As pre-registered, we regressed participants’ judgments against judgment type (“think”/“say”) and power level (“popular”/“anonymous”), as well as a random intercept for participant × speaker (rating~judgmentType*powerLevel+(1|subID:SpkrID)). Participants in both conditions expected informants to continue to privately believe Blue, but as predicted, Popular condition participants expected private beliefs to shift more toward Yellow than Anonymous condition participants (*β*_popularBelief_Intercept_ = −4.95, *SE* = .25, *p* < .001, *β*_anonBelief_ = −1.39, *SE* = .35, *p* < .001). Also as predicted, participants expected informants’ shift toward the Yellow design to be greater for public votes than private beliefs in the *Popular* condition (*β*_popularVote_ = 6.22, *SE* = .25, *p* < .001), and the difference between public vote and private beliefs was smaller in the Anonymous condition (*β*_anonVote_Intxn_ = −3.86, *SE* = .35, *p* < .001). The result of these differently-sized shifts toward Yellow was an expectation of pluralistic ignorance in the *Popular* condition but not the *Anonymous* condition: on average, *Popular* condition participants expected informants to publicly vote Yellow even though they privately believed Blue (M_avgVote_centered_ = 0.09, *t*(117, 1) = 3.49, *p* < 0.001; M_avgBelief_centered_ = −0.22, *t*(117, 1) = −10.4, *p* < 0.001), whereas *Anonymous* condition participants expected informants to endorse Blue both publicly and privately (M_avgVote_centered_ = −0.17, *t*(119, 1) = −7.21, *p* < 0.001; M_avgBelief_centered_ = −0.29, *t*(119, 1) = −16.1, *p* < 0.001).

**Figure 2.**
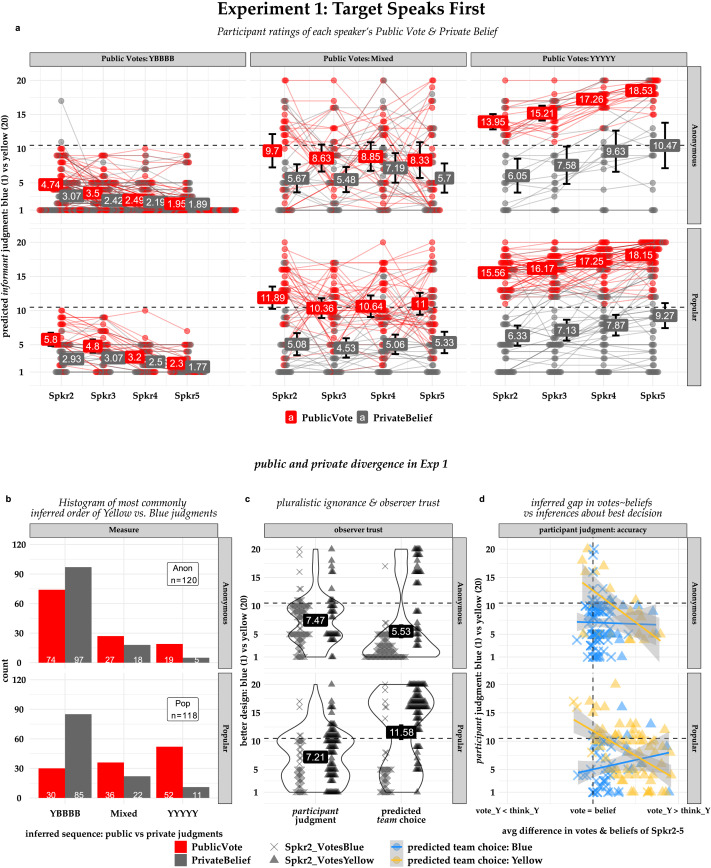
Experiment 1 results. **Panel 2a:** Inferred confidence for each speaker’s public vote and private belief; values denote means, lines join each participant’s ratings. **Panel 2b:** Histogram of predicted vote and belief sequences. For visualization, participants are grouped by their predicted vote sequence: YYYYY (“all speakers will vote (or believe) yellow”), YBBBB (“only Spkr_1 will vote (or believe) yellow”), and “Mixed” (all other sequences). **Panel 2c:** Violin plots & mean ratings for which design the participant themselves believes is best, and which design they expect the team choose in the post-sequence discussion. **Panel 2d:** Participant judgment of best design (*y*-axis) versus the average difference in their predictions about informants’ votes versus beliefs (*x*-axis), with participants grouped (color) by their predictions about the team’s choice in the post-sequence discussion and Spkr2’s vote (shape).

As shown in Figure 2b, roughly 75% of participants in both conditions expected all four subsequent speakers to continue to privately believe blue. But whereas 88 out of 118 of *Popular* condition participants expected one or more public votes to flip to yellow—and 52 of those expected all four votes to flip to yellow—only 46 of 120 *Anonymous* condition participants expected one or more public votes to flip, and nearly as many participants expected only one vote to flip (15) as all four (19).

And as shown in Figure 2a, for participants who expected speakers 2–5 all to vote the same way (i.e., YYYYY, YBBBB) the vote pattern each participant predicted was systematically related to the way their confidence changed during the vote sequence; but this wasn’t the case for participants who predicted a mixed pattern of votes (Mixed, e.g., YYBBB, YBYBB). In both conditions, participants who predicted a YYYYY vote pattern were increasingly certain later speakers would *vote* Yellow (**Pop_YYYY**: *β*_vote_Intercept_ = 4.95, *SE* = .26, *p* < .001, *β*_vote_SpkrOrder_ = 0.89, *SE* = .10, *p* < .001; **Anon_YYYY**: *β*_vote_Intercept_ = 3.37, *SE* = .42, *p* < .001, *β*_vote_SpkrOrder_ = 1.58, *SE* = .14, *p* < .001), and *less* certain they would continue to *believe* Blue (**Pop_YYYY**: *β*_believe_Intercept_ = −4.28, *SE* = .77, *p* < .001, *β*_believe_SpkrOrder_ = 0.96, *SE* = .16, *p* < .001; **Anon_YYYY**: *β*_believe_Intercept_ = −4.36, *SE* = 1.34, *p* = .004, *β*_believe_SpkrOrder_ = 1.53, *SE* = .26, *p* < .001). Similarly, participants who predicted a YBBBB vote pattern were increasingly certain later speakers would both *vote* and *believe* Blue (**Pop_YBBBB**: *β*_vote_Intercept_ = −4.66, *SE* = .39, *p* < .001, *β*_vote_SpkrOrder_ = −1.21, *SE* = .12, *p* < .001; **Pop_YYYY**: *β*_believe_Intercept_ = −7.32, *SE* = .31, *p* < .001, *β*_believe_SpkrOrder_ = −0.41, *SE* = .08, *p* < .001; **Anon_YBBB**: *β*_vote_Intercept_ = 5.92, *SE* = .24, *p* < .001, *β*_vote_SpkrOrder_ = −0.94, *SE* = .08, *p* < .001; *β*_believe_Intercept_ = −7.54, *SE* = .23, *p* = .004, *β*_believe_SpkrOrder_ = −0.38, *SE* = .08, *p* < .001). But, participants who predicted a mixed pattern were no more confident about either the votes or beliefs of later speakers compared to earlier speakers (see supplemental materials Figure SI.3 for visualization of coefficients). In other words, participants’ confidence ratings seemed to reflect an intuitive understanding of information cascades consistent with the Bobcat Bite scenario; we’ll return to this point in the preregistered analysis of our computational model results that the non-preregistered regression above supplements.

As shown in Figure 2c, participants expected the team’s final choice to confirm their pre-sequence “Blue” judgments in the Anonymous condition (*β*_anon_centerIntercept_ = −4.97, *SE* = .26, *p* < .001) but endorse the dissenter’s “Yellow” judgment in the Popular condition (*β*_pop_centerIntercept_ = 1.08, *SE* = .36, *p* < .001), in both cases following the number of *public* votes they expected that design to receive during the sequence itself (*β*_anon_scale(PubVoteMaj)_ = 5.17, *SE* = .26, *p* < .001; *β*_pop_scale(PubVoteMaj)_ = 5.38, *SE* = .36, *p* < .001). However, when we asked which design they themselves believed was best, participants in both conditions were equally confident in the design endorsed by the pre-sequence consensus (M_anon_ = −3.02, *t*(119, 1) = −7.88, *p* < .001; M_pop_ = −3.29, *t*(117, 1) = −7.94, *p* < .001). Figure 2d shows how participants’ own judgments about which design was best was related to their expectation that their informants would publicly vote for a design they didn’t privately believe in. As pre-registered, we centered ratings on the midpoint of the scale (10.5), and averaged each participants’ predictions about the informants’ public votes and private beliefs. Our prediction was that our “model A” (trustMaj ~ inferAvg_PrivateBeliefs) would outperform any model based on PublicVotes, condition, or their combination, reasoning that neither public votes nor the dissenter’s popularity would elicit any greater trust in the majority judgment. This was only partially borne out—public votes and popularity improved the fit, but *decreased* trust. Further exploratory analyses showed that for participants in both conditions who expected the team’s final decision to contradict the pre-sequence consensus, trust in that decision declined as the *gap* they expected to find between the informants’ votes and beliefs increased (*β*_anon_centerIntercept_chooseYellow_ = 2.22, *SE* = 1.21, *p* = 0.07, *β*_anon_diffVoteBelief_chooseYellow_ = −0.56, *SE* = 0.15, *p* < 0.001; *β*_pop_centerIntercept_chooseYellow_ = 1.32, *SE* = 0.83, *p* = 0.11, *β*_pop_diffVoteBelief_chooseYellow_ = −0.44, *SE* = 0.09, *p* < 0.001). But, the same wasn’t true of participants who expected the team’s final decision to endorse the pre-sequence consensus (*β*_anon_chooseBlue_ = −5.63, *SE* = 1.30, *p* < 0.001, *β*_anon_diffVoteBelief::chooseYellow_ = 0.54, *SE* = 0.21, *p* = 0.01; *β*_pop_chooseBlue_ = −6.79, *SE* = 1.11, *p* < 0.001, *β*_pop_diffVoteBelief::chooseYellow_ = 0.61, *SE* = 0.15, *p* < 0.001). So on the one hand, most participants expected the team’s final decision to confirm whichever design the majority had publicly voted for during the sequence, regardless of whether they thought the informants’ privately believed in it. But on the other hand, participants themselves trusted the design they thought their informants privately believed in, regardless of whether they expected the informants to publicly vote for it.

#### Computational Model Fits.

**Figure 3.**
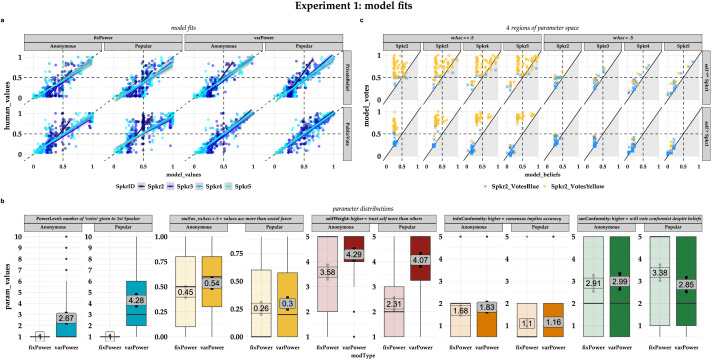
**Panel 3a**: Model fits for participants’ inferences about each speaker’s public and private judgments in each condition of Experiment 1. **Panel 3b:** Distribution of optimal fitting parameters for participants in each condition of Experiment 1 according to the fixedPower (faded) and varPower model (dark). **Panel 3c**. In the varPower model of Experiment 1, predictions about Spkr2’s vote are propagated through the remainder of the sequence in qualitatively different ways for different regions of the parameter space defined by whether or not accuracy outweighs social favor (*ω*_acc_ > .5 versus < = .5) and whether or not informants’ own beliefs outweigh Spkr1’s vote (W_Self_ > W_Power_ versus W_Self_ < = W_Power_). The diagonals divide predictions that favor Yellow more strongly publicly than they do privately (upper, unshaded), or vice versa (lower, shaded).

To better understand participants’ intuitions about social favor, and how these intuitions drove their predictions, we fit each of our generative models to each participant’s responses individually. Figure 3a compares participants’ inferences about public and private judgments in both conditions with model predictions from both the full varPower model (which allowed the weight of the Yellow informant’s vote to vary by participant between 0 and 10) and the lesioned fixedPower model (which fixed the weight of every informant’s vote at 1). The varPower model fits were higher than the fixedPower fits for either condition (Anonymous: R_varPower_ = 0.89, 95CI: [0.88–0.90], R_fixPower_ = 0.83, 95CI: [0.81–0.85]; Popular: R_varPower_ = 0.92, 95CI: [0.91–0.93], R_fixPower_ = 0.80, 95CI: [0.77–0.82]), and as predicted, a bootstrapped 95% confidence interval over the difference in correlation revealed that the varPower model outperformed the fixedPower model by a greater margin in the Popular condition (95CI: [.10, .14]) than in the Anonymous condition (95CI: [.04, .07]).

A glance at the slopes Figure 3a is enough to see that Spkr2 stands out as considerably steeper than the rest: regardless of which design participants expected Spkr2 to endorse, neither model’s predictions about Spkr2’s private beliefs were as extreme as participants’ were—and for the public vote predictions, only the varPower model matched participants’ confidence as well for Spkr2 as it did for later speakers. Why? Because by design, the *first* speaker always voted Yellow. So while both models predicted each speaker’s public and private judgments using whatever sum of votes a participant had inferred thus far, those sums varied by participant for Spkr3-5; but for Spkr2, the models used the same 1–0 sum for every participant. However, the W_Power_ parameter in the varPower model was able encode the disproportionate influence that some participants expected Spkr1 to have when predicting Spkr2’s public vote without forcing a corresponding influence on Spkr2’s private beliefs, allowing it to capture participants’ expectations about how a popular first speaker might lead subsequent informants’ public and private beliefs to diverge. In other words, the varPower model may have outperformed the fixedPower model because being able to use W_Power_ to better predict Spkr2’s vote allowed it to better predict later speakers’ judgments as well.

Comparing the by-condition differences in optimal fits from the fixedPower and varPower model (Figure 3b) suggests the same story. Unsurprisingly, the varPower model fits allowed the Yellow dissenter’s vote to carry more weight in the *Popular* condition than in the *Anonymous* condition (**W**_**Power**_: M_Pop_varPwr_ = 4.28, M_Anon_varPwr_ = 2.67, *t*(230) = −4.33, *p* < 0.001, 95CI: [2.33–0.87]). And both models predicted that (1) social favor would outweigh accuracy more in the *Popular* condition than the *Anonymous* condition (***ω***_**acc**_: M_Pop_varPwr_ = 0.30, M_Anon_varPwr_ = 0.54, *t*(236) = 5.85, *p* < 0.001, 95CI: [0.16–0.32]; M_Pop_fixPwr_ = 0.26, M_Anon_fixPwr_ = 0.45, *t*(236) = 4.46, *p* < 0.001, 95CI: [0.11–0.28]), and that (2) participants would expect their informants to adjust their *private beliefs* less in response to numerically stronger majorities in the *Popular* condition than the *Anonymous* condition (***θ***_**infoCon**_: M_Pop_varPwr_ = 1.16, M_Anon_varPwr_ = 1.83, *t*(210) = 4.57, *p* < 0.001, 95CI: [0.38–0.96]; M_Pop_fixPwr_ = 1.10, M_Anon_varPwr_ = 1.68, *t*(217) = 4.01, *p* < 0.001, 95CI: [0.29–0.86]). Significant main effects in ANOVAs comparing model fits by condition also suggested that social favor outweighed accuracy more strongly in both conditions of the fixedPower model than the varPower model (***ω***_**acc**_: F(236, 1) = 8.81, *p* = 0.003) but that later speakers’ private beliefs were no more more deferent to numerically larger majorities according to the fixedPower model than the varPower model (***θ***_**infoCon**_: F(236, 1) = 9.10, *p* = 0.003).

However, we also found significant interactions in ANOVAs comparing model fits by condition for informants’ trust in their initial judgment (W_Self_: F(236, 1) = 37.2, *p* < 0.001), and their willingness to adjust their public votes in response to a numerically stronger majority of weighted votes (***θ***_**socCon**_: F(236, 1) = 4.70, *p* = 0.031). These parameters didn’t differ in the varPower model (**W_Self_**: M_Pop_ = 4.07 versus M_Anon_ = 4.29, *t*(233) = 1.24, *p* = 0.217, 95CI: [−0.13–0.58], and ***θ***_**socCon**_: M_Pop_ = 2.85 versus M_Anon_ = 2.99, *t*(234) = 0.56, *p* = 0.578, 95CI: [−0.36–0.64]), but were significant or approaching in the fixedPower model (**W**_**Self**_: M_Pop_ = 2.31 versus M_Anon_ = 3.58, *t*(232) = 6.21, *p* < 0.001, 95CI: [0.87–1.67], and ***θ***_**socCon**_: M_Pop_ = 3.38 versus M_Anon_ = 2.91 for *t*(236) = −1.75, *p* = 0.081, 95CI: [−1.00–0.06]).

That analysis suggests an important difference in the intuitive theories encoded by our two models, and how two different observers with each theory would interpret the same predictions. The fixedPower model reflects an observer who assumes that all speakers have equal social status. The only way for such an observer to explain Spkr1’s disproportionate effect on Spkr2 in the Popular condition is to assume that Spkr2 has very little confidence in their own opinion (i.e.: decreasing W_Self_), and/or assume that Spkr2 is very reactive to social consensus (i.e., increasing *θ*_socCon_). But since the model fits each participant with one set of parameters for every informant, adjusting these assumptions makes *every* speaker more likely to vote with previous speakers regardless of their private beliefs, and amplifies the effect for later speakers by making their deference to the majority increase with its numerical advantage over the minority opinion. This highlights the varPower model’s advantage in explaining participants’ judgments: an observer who assumes Spkr1 might have disproportionate social status (i.e.: higher W_Power_ relative to other voters) can first detect whether or not W_Power_ outweighs W_Self_ by enough to make Spkr2 vote against their beliefs, and then propagate that influence through the sequence in the form of the changing sum of votes that each speaker weighs against their own beliefs and desire for social favor.

Figure 3c shows how the varPower model propagates participants’ predictions about Spkr2’s vote through the remainder of the sequence by zooming in on four different regions of the parameter space in which the model makes qualitatively different predictions. These four regions are defined by whether or not participants expect accuracy to outweigh social favor or vice versa (*ω*_acc_ > 0.50), and whether or not they expect the informants’ own initial judgment to outweigh the first speaker’s vote (W_Self_ > W_Power_).

In Region 1 (top row, left set of columns), participants expect informants to value social favor at least as much as accuracy, and expect Spkr1’s vote to carry at least as much weight as the informants’ own beliefs. In this case, they almost exclusively predict that Spkr2 will vote Yellow, and predictions for every speaker lie above the diagonal, indicating that participants are more confident that informants will endorse Yellow publicly than privately. However, while participants are no more confident that later speakers will publicly *vote* Yellow, they are more confident that later speakers will come to privately *believe* Yellow. Intuitively: when informants have little trust in their own beliefs and don’t value accuracy more than social favor, participants believe that a powerful first voter may be able to genuinely turn consensus in their favor by triggering an information cascade.

In Region 2 (bottom row, left set of columns), participants also expect informants to value social favor at least as much as accuracy, but they expect informants own beliefs to outweigh Spkr1’s vote. In this case, participants are divided about whether Spkr2 will vote Blue or Yellow, but their predictions about Spkr2’s vote appear to cascade through the sequence in different ways. Those participants who expect Spkr2 to vote Yellow not only appear to be increasingly confident that each successive speaker will *vote* Yellow; they also expect each successive speaker’s private *beliefs* to shift towards Yellow—though once again, the distribution of points above the diagonal indicates they are still more confident that informants will endorse Yellow publicly than privately. For participants who expect Spkr2 to vote Blue, the pattern is reversed: they are increasingly confident that both public and private judgments will shift toward Blue with each successive speaker. Intuitively: if informants’ own beliefs outweigh Spkr1’s vote but accuracy doesn’t outweigh social favor, participants think Spkr2’s vote is pivotal—a Blue vote lets other informants vote their beliefs as well, while a Yellow vote makes them vote Yellow even if they privately believe Blue.

In Regions 3 and 4 (right set of columns, bottom row and top row, respectively), accuracy outweighs social favor. Notably, Region 4 is almost empty: few participants in our experiment who expected accuracy to outweigh social favor also expected informants to give more weight to Spkr1’s vote than their own beliefs—but those who did also expected informants’ public and private judgments to converge. In Region 3, participants who expected informants’ own beliefs to outweigh Spkr1’s vote almost exclusively predict Spkr2 will vote Blue, and are again increasingly confident that each successive speaker’s public and private judgments will shift further toward Blue. Intuitively: when informants value accuracy more than social favor, participants expect informants to either vote their beliefs (if their beliefs outweigh Spkr1’s vote), or come to believe in whatever the consensus votes (if Spkr1’s vote outweighs their beliefs).

The gist: participants thought informants were unlikely to vote against their initial beliefs unless social favor outweighed accuracy *and* Spkr2 endorsed Spkr1’s Yellow vote (352 of 412 predicted Yellow votes come when *ω*_acc_ < = 0.5 and Spkr2 votes Yellow). But, they mainly expected Spkr2 to vote against the Spkr1’s Yellow vote if they thought the informants’ W_Self_ outweighed Spkr1’s W_Power_ (408 of 508 predicted Blue votes from Spkr2 come when W_Power_ < W_Self_). Overall, this meant that participants expected larger gaps between their informants’ public votes and private beliefs when W_Power_ outweighed W_Self_ than vice-versa. And the greater that gap, the *more* they expected the team to choose the dissenter’s design after the post-sequence discussion and the *less* likely they were to endorse that design themselves (Figure 2c, Figure SI.4). The different intuitions in these four regions not only help explain why people might come to qualitatively different conclusions about how the first speaker’s vote will influence subsequent speakers; they also help explain whether or not they treat the final consensus judgment as reliable.

## EXPERIMENT 2

Consensus strength among independent informants can be evidence of decision quality (Kameda et al., 2012, 2022) in the same way that rolling more 6s can be stronger evidence of weighted dice—except that our real-world informants’ beliefs, unlike rolls of the dice, are rarely independent in the necessary sense (Eriksson et al., 2007; Laan et al., 2017). Fortunately, our real-world informants are also better than dice at evaluating each other’s knowledge (Aboody et al., 2024; Harris et al., 2018; Hawkins et al., 2023; Navajas et al., 2018; Rendell et al., 2010), meaning that in some cases, their consensus may be evidence of decision quality even when their judgments are not independent. The trick is distinguishing reliable consensus from unreliable consensus, regardless of whether it’s independent or not.

Participants in Experiment 1 clearly realized that public consensus can snowball in a way that reflects their informants’ desire for social favor more than their genuine beliefs about the best design; and no matter how many public votes they expected that snowball to gather, participants themselves endorsed the design initially favored by the *private* consensus. But real life doesn’t give people the opportunity to compare their teammate’s private beliefs and public votes. Still, a stronger-than-expected consensus does often seem to waggle its brow and gesture furtively at any hint of an ulterior motive (Bhui & Gershman, 2020; Gunn et al., 2016; Mann, 2024; Munroe, n.d.). Thus, in Experiment 2, we ask whether a unanimous public consensus would elicit more skepticism if it followed a Popular first speaker than an Anonymous first speaker, even if we don’t give participants any information about the informants’ private beliefs.

### Participants

We again aimed to recruit 120 respondents per condition (Popular or Anonymous) from mTurk, and after screening out *n* = 19 mTurkers prior to participating for having failed basic comprehension checks, we were left with a final sample of *n* = 123 in each condition.

### Procedure

As in Experiment 1, participants were introduced to a five-person team building a remote control airplane for a contest using one of two designs. But instead of showing them the teammates’ initial private beliefs about which design was best, as in Experiment 1, participants in Experiment 2 were simply introduced to the first speaker (again either omitting mention of his status or calling him “Max” and describing him as “very popular”), and shown that, one-by-one, each speaker publicly voted yellow. Participants in Experiment 2 then rated which airplane they themselves believed was best, and predicted which design the team would choose, briefly explaining their answers for each. Participants responded using a 20-point scale anchored at 1-“definitely blue” and 20-“definitely yellow”.

### Results and Discussion

**Figure 4.**
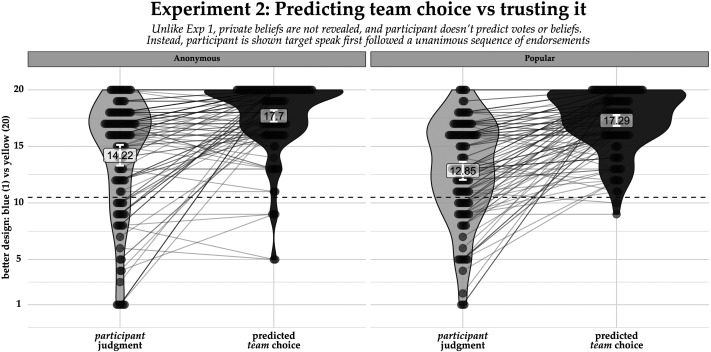
Participant predictions about the team’s final decision after the post-sequence discussion (black) and which design they themselves believed was actually best (grey), depending on whether the first speaker was Popular or Anonymous (facets). *Y*-axis values indicate propensity to endorse yellow (high values) or blue (low values).

In order to compare participants’ inferences about which design the team would choose after discussing and which design was actually best, we centered the response scale at the midpoint (10.5) of the 20-point scale, and regressed participants’ judgments against the first speaker’s power level (trustMaj-10.5~PowerLevel), as preregistered. As shown in Figure 4, participants in both conditions were equally confident that the team would choose the Yellow design (*β*_Intercept_anon_ = 7.20, *SE* = .26, *p* < .001, *β*_Popular_ = −0.407, *SE* = .36, *p* = 0.266). Participants in both conditions also believed that the Yellow design was superior to the Blue design (*β*_Intercept_anon_ = 3.72, *SE* = .43, *p* < .001, *β*_Intercept_pop_ = 2.35, *SE* = .43, *p* < .001). As predicted, however, confidence ratings were significantly lower when the first speaker was Popular than Anonymous (*β*_PwrCondition_popular_ = −1.37, *SE* = .61, *p* < .026). In other words, participants don’t need access to their informants’ private beliefs to be more suspicious of a sequence of votes that endorses a Popular first speaker than one that endorses an Anonymous first speaker.

## EXPERIMENT 3

Our variable power model attempts to capture the effects of speaking order and popularity by making informants’ judgments depend on the sum of votes for each design but allowing the each vote’s weight to vary. But in Experiments 1 and 2, the dissenter is always the first speaker; we only manipulate their popularity. In Experiment 3, we reverse the speaking order, so that the dissenter’s chance to speak only comes after everyone else has voted. Since beliefs are private and the first speaker always votes their beliefs, this means starting the sequence with a Blue vote. This design allows us to ask whether participants believe that (1) a dissenter’s influence over a sequential vote depends on their speaking order as much as their popularity, (2) a popular dissenter may be more steadfast in their judgment (either private or public) than an anonymous dissenter, and (3) a popular dissenter may have enough power to flip the team’s final decision by coming out for yellow even if the private consensus for blue had already become public.

### Participants

As in Experiment 1, we aimed for a final sample of 120 respondents per condition (Popular or Anonymous) from mTurk. After screening out n = 15 mTurkers prior to participating for failing basic attention checks and one for a bot-like explanation, our final sample included n = 121 in the Anonymous condition and n = 120 in the Popular condition.

### Procedure

The procedure was identical to Experiment 1, except that speaking order was reversed. This meant that (1) having the first speaker vote their beliefs would start the sequence with a blue vote, and (2) the one teammate who privately believed yellow at the outset—“Max” in the Popular condition, unnamed in the Anonymous condition—wouldn’t have a chance to speak until the other four teammates (who all privately believed blue initially) had already voted. As in Experiment 1, we asked participants to infer the public vote and private belief of speakers 2–5, assuming the sequence of votes thus far was as they predicted. Also as in Experiment 1, we said that after voting, the contest organizers asked the team to talk together to make a final decision, and asked participants to infer (1) which design the team would settle on, and (2) which design they themselves thought was best. Participants responded to all questions using a 20-point scale anchored at 1-“definitely blue” and 20-“definitely yellow”; for the final-team-decision and true-best questions, they were also asked to briefly explain their answers.

### Results and Discussion

**Figure 5.**
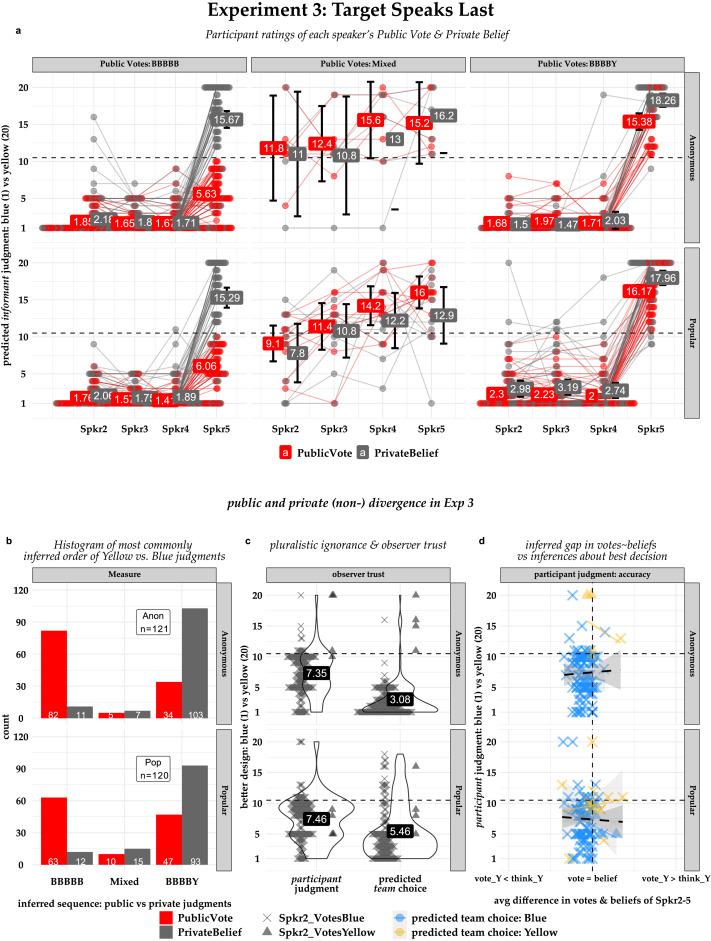
Experiment 1 results. **Panel 5a:** Inferred confidence for each speaker’s public vote and private belief; values denote means, lines join each participant’s ratings. **Panel 5b:** Histogram of predicted vote and belief sequences. For visualization, participants are grouped by their predicted vote sequence: BBBBB (“all speakers will vote (or believe) blue”), BBBBY (“all but Spkr5 will vote (or believe) blue”), and “Mixed” (all other sequences). **Panel 5c:** Violin plots & mean ratings for which design the participant themselves believes is best, and which design they expect the team choose in the post-sequence discussion. **Panel 5d:** Participant judgment of best design (*y*-axis) versus the average difference in their predictions about informants’ votes versus beliefs (*x*-axis), with participants grouped (color) by their predictions about Spkr2’s vote (shape), but collapsing the separate fits for the team’s predicted final decision after the post-sequence discussion (color, faded) into a single fit (black, dashed) because nearly all participants’ predicted the same final decision.

We ran the same regression model from Experiment 1, but separately for Spkr5 and Spkr2-4, because reversing the speaking order meant that Spkr5’s initial private beliefs now differed from Spkr2-4’s. As predicted and in contrast to Experiment 1, participants in the Popular condition were confident that Spkr2-4 would endorse Blue both publicly *and* privately (*β*_popularBelief_Intercept_ = −7.48, *SE* = .17, *p* < .001, *β*_popularVote_ = −0.37, *SE* = .13, *p* = .004). On average, Anonymous condition participants were slightly more confident than Popular condition participants about Spkr2-4’s private beliefs, but not about their public votes (*β*_anonBelief_ = −0.78, *SE* = .24, *p* = .001; *β*_anonVote_Intxn_ = 0.35, *SE* = .18, *p* = 0.053). As shown in Figure 5b, approximately 50% of Popular and 70% of Anonymous condition participants expected Spkr5 to publicly vote Blue, even though almost all participants in both conditions expected Spkr5 to continue to privately believe Yellow and Spkrs2-4 to endorse Blue both publicly and privately.

And as shown in Figure 5a, a sharp discontinuity between Spkr2-4 and Spkr5 for all but a fraction of participants replaced the gradual shift in confidence with each successive speaker observed in Experiment 1. Participants in both conditions were equally confident that the dissenting 5th speaker would continue to believe Yellow privately (*β*_popularBelief_Intercept_ = 5.63, *SE* = .47, *p* < .001; *β*_anonBelief_ = 0.29, *SE* = .67, *p* = 0.67). But their expectations about the 5th speaker’s public vote shifted significantly toward Blue in the Popular condition, and shifted even more in the Anonymous condition (*β*_popularVote_ = −5.28, *SE* = .55, *p* < .001; *β*_anonVote_Intxn_ = −2.37, *SE* = .77, *p* = 0.002), suggesting that participants in the Popular condition were more confident that a private dissenter who spoke last would steadfastly resist conformist pressure.

Finally, Figures 5c–d suggest that in contrast to Experiment 1 (Figure 3c–d), participants in Experiment 3 were not concerned that informants’ public votes were less reliable evidence than their private beliefs about which design was better. As in Experiment 1, we centered ratings on the midpoint of the scale (10.5), and averaged the each participants’ predictions about the informants’ public votes and private beliefs. And as in Experiment 1, participants’ predictions about which design the *team* would endorse during the post-sequence discussion followed the number of *public* votes they expected that design to receive during the sequence itself in both conditions (*β*_anon_scale(PubVoteMaj)_ = 2.47, *SE* = .18, *p* < .001; *β*_pop_scale(PubVoteMaj)_ = 2.42, *SE* = .34, *p* < .001). But unlike in Experiment 1, almost all participants in *both* conditions (102 of 120 Anonymous and 116 of 121 Popular) expected the team’s final choice to confirm their pre-sequence “Blue” judgments (*β*_anon_centerIntercept_ = −7.42, *SE* = .18, *p* < .001; *β*_pop_centerIntercept_ = −5.04, *SE* = .34, *p* < .001). And participants were just as confident in both conditions of Experiment 3 as they were in Experiment 1 that the design favored by the pre-sequence majority was best (M_anon_ = −3.15, *t*(120, 1) = −9.07, *p* < .001; M_pop_ = −3.04, *t*(119, 1) = −8.96, *p* < .001). Because so few participants in either condition expected the team’s final decision to be Yellow and the dissenter’s popularity had no effect on participants’ trust in the team’s final decision, we collapsed across PowerLevel to test the effect of belief-vote gaps on participants’ trust in the team’s decision. Participants who expected a Blue final team decision were more confident that Blue was the better design than those who expected a Yellow final team decision (*β*_centerIntercept_Blue_ = −5.28, *SE* = .25, *p* < .001, *β*_decisionYellow_ = 7.36, *SE* = .59, *p* < .001), but, unlike in Experiment 1, neither group’s confidence in the team’s final decision was related to the size of the average vote-belief gap they expected in their informants’ judgments (*β*_avgGap_Blue_ = 0.05, *SE* = .09, *p* = 0.598, *β*_avgGap:decisionYellow_ = 0.0005, *SE* = .20, *p* = 0.998).

#### Computational Model Fits.

By construction, the within-condition fits for the varPower model were identical to the fits for the fixedPower model in Experiment 3. This is because making the dissenter speak last nullifies the only difference between the models—namely, that the varPower model allows the influence the dissenter’s vote has on *subsequent* speakers (W_Power_) to vary, but the fixedPower model fixes it at 1.00 along with the rest of the informants. The main question, then, is whether the now-identical models performed better in one condition than the other. Per our pre-registered hypothesis, we expected the varPower model to outperform the fixedPower model only when the private dissenter both a) spoke first and b) was identified as popular, and thus expected no significant difference in model fit across conditions for Experiment 3. What we found instead was that the models performed slightly but significantly better in the Anonymous condition than the Popular condition (R_anon_ = .89, 95CI: [.88, .91]; R_pop_ = .86, 95CI: [.84, .88]). The most likely explanation for this difference is that (as noted in the regression analysis above) participants were more likely to infer that the dissenter would vote his beliefs in the Popular condition than the Anonymous condition. That is, participants expected a Popular dissenter to have more confidence in their own beliefs (i.e., higher W_Self_). However, our models weren’t able to capture that intuition, for approximately the same reason that the fixedPower model underperformed the varPower model in Experiment 1: there’s only one parameter (W_Power_) that can adjust predictions for a Popular dissenter without inflating error for the rest of the informants, and making the dissenter speak last nullifies it for both models in Experiment 3 just as much as fixing it at 1 nullifies it for the fixedPower model in Experiment 1.

## GENERAL DISCUSSION

Let’s recap. The problem with sequential voting is that if Alice’s informants use their total (i.e., independent + testimonial) evidence to make their judgments, then at some point enough testimonial evidence will accumulate that her informants will start endorsing the consensus judgment even if it contradicts their independent evidence. Once that happens, the Condorcet-adjacent algorithms that justify Alice’s treating stronger consensus as signaling stronger evidence start getting unreliable. But *when* it happens depends on how much weight her informants put on their own independent evidence relative to testimony. And that’s the key insight. If Alice doesn’t know when testimony will start outweighing her informants’ independent evidence, worrying about Condorcetian independence might have her trusting sequential consensus too *little* instead of too much.

By fitting a parameterized computational model to participant judgments, we were able to estimate what people think those relative weightings are, and how they combine with non-epistemic factors to shape informants’ judgments in sequential voting processes. We did this by showing people scenarios that either maximize or minimize the potential impact of sequential voting: a 1v4 consensus of private judgments in which the dissenter votes either first or last, and is either described as “very popular” or not distinguished from the rest. The model posited that participants estimate each speaker’s vote using a three step process. First, the speaker updates their private *beliefs* in proportion to the sum of unweighted votes for each option, modulated by the informational value of consensus (*θ*_infoCon_) and their degree of confidence in their own initial beliefs (W_Self_). Second, the speaker computes the social favor to be gained by voting with consensus (*θ*_socCon_) using the sum of votes weighted by the voter’s popularity (W_Power_), which is held equal for all speakers in the fixedPower model, but allowed to vary for the popular speaker in the varPower model. Finally, the speaker decides how to vote by weighing their desire to vote accurately against their desire for the social favor that could be gained by voting with a particular side (***ω***_**acc**_).

Simulating the varPower model’s parameter space for our scenario suggested that because speaking last prevents the dissenter’s popularity from having any effect at all on previous speakers, he’ll end up facing a strong Blue consensus that will make him almost certain to conform regardless of his popularity (Figure SI.2). But speaking first (Figure SI.1) often allows him to overturn the private consensus entirely, especially when his popularity outweighs the informants’ trust in their own initial judgment (W_Self_ < = W_Power_), or the informants don’t value accuracy more than social favor (i.e., ***ω***_**acc**_ < = .50). The simulation also suggested that allowing the dissenter to speak first makes Spkr2’s vote pivotal: a Yellow vote from Spkr2 nearly guarantees a unanimous consensus regardless of the other parameter values, while a Blue vote from Spkr2 frequently keeps any other subsequent speaker from voting Yellow either unless social favor outweighs accuracy (*ω*_acc_ < = 0.50) or the dissenter’s power outweighs the informants’ initial judgment (W_Self_ < = W_Power_).

Our participants seemed to share those insights. When the dissenter spoke first, nearly 75% of them (177 of 241) expected Spkr3-5 to *all* vote whichever way Spkr2 did—which was usually Yellow if Spkr1 was Popular (78 of 120 in Popular condition) and Blue if Spkr1 was Anonymous (86 of 121 in Anonymous condition). In contrast, only 15% of participants expected anyone but the dissenter to vote Yellow when he spoke last—though participants did expect the dissenter himself to be more steadfast in his beliefs in the Popular condition (56 of 120 predicted a Yellow vote) than the Anonymous condition (44 of 122 predicted a Yellow vote). But as predicted, most participants in both experiments (~76% in Exp 1 and ~91% in Exp 3) expected *all* the informants to continue to believe Blue privately, albeit with less confidence when the dissenter (especially a Popular one) spoke first. Also as predicted, the varPower model outperformed the fixedPower model more strongly in the Popular condition of Experiment 1 than any of the other three conditions in Experiments 1 or 3—in part because parameterizing the dissenter’s non-epistemic influence (W_Power_) separately from the informants’ desire for accuracy (*ω*_acc_) allowed the model to predict Spkr1’s disproportionate influence on Spkr2’s vote without causing it to overestimate later speakers’ probability of voting Yellow (e.g., especially when Spkr2 voted Blue). Our results therefore support the conclusion that participants similarly recognize the influence of Spkr 1’s social power separately from speakers’ general disposition towards accuracy versus social favor.

All of that tells the same story: manipulating social favor made participants more likely to think their informants would publicly vote for a design they didn’t privately believe in. But it didn’t make them *trust* those votes, as you might have expected from past work. However, participants in those studies would have had no cause for suspecting that their informants’ public votes might contradict their private evidence unless they happened to correctly compute the conditional probabilities that a Bayes-normative informant would face at each step in the sequence (Whalen et al., 2018; Xie & Hayes, 2022). In contrast, participants in every condition of Experiment 1 and 3 were equally confident in the design endorsed by the pre-sequence consensus—because the greater the gap they expected to find between the informants’ public and private judgments, the less they trusted a final team decision that contradicted it (Figure 2c, Figure 5c). And in Experiment 2, participants were more skeptical of a unanimous sequence when the first speaker was Popular than when he was Anonymous, even though we hadn’t shown them the pre-sequence consensus or prompted them for separate predictions about how informants’ public and private judgments would change. In other words, participants trusted consensus to the extent that they thought it reflected their informants’ best judgment; and social favor made them doubt that it did, even if they had no other evidence about the informants’ private beliefs.

Our computational model gives a more detailed picture of participants’ reasoning, highlighting in particular their understanding that the second speaker’s vote is pivotal (consistent with the seminal work from Bikhchandani et al., 1992) as well as their beliefs about why the informants’ private beliefs might be more reliable evidence than their public votes. Two intuitions divide the model’s parameter space into four distinct regions that help explain how the sequence amplifies early votes: whether or not participants expected a non-epistemic value like social favor to carry as much weight as the informants’ (1) own initial judgment or (2) desire for accuracy. In general, participants who gave more weight to social favor were also more likely to expect more votes to flip during the sequence, and predict a final team decision that contradicted the pre-sequence consensus. Both intuitions depended on the dissenter’s speaking order and popularity, but they played slightly different roles (Figure 3c): high self weight (W_Self_ > W_Power_) made informants’ private beliefs resistant to the first speaker’s vote, but low accuracy values (*ω*_acc_ < = 0.5) made their public votes track the sum of previous speakers’ votes instead of their private beliefs. Those two intuitions together made Spkr2’s vote pivotal: any value of *ω*_acc_ that was too low to make Spkr2 publicly contradict Spkr1 was even less likely to make Spkr3 contradict their two votes together, even if W_Self_ was high enough to keep the informants’ private beliefs from following their public votes. So, almost everyone expected *all* of the informants to vote whichever way Spkr2 voted, and very few participants expected Spkr2 to publicly contradict Spkr1 unless the informants’ initial beliefs outweighed the dissenter’s vote. Everyone else not only expected Spkr2’s vote to flip, but expected every other informants’ votes and beliefs to follow. So in the end, participants only expected the informants’ public votes to misrepresent the pre-sequence consensus if a dissenter spoke first *and* was popular enough to flip Spkr2’s vote.

A caveat: design differences mean our results can’t be compared directly to past work. Past work (Whalen et al., 2018; Xie & Hayes, 2022) didn’t show participants the informants’ pre-sequence judgments; it just pushed them to treat those judgments as tantamount to ground truth by giving each informant the kind of statistical evidence that participants would justifiably consider unmistakeable for any adult human (a ball from urns with opposite 80–20 proportions). Past work also didn’t prompt participants to make separate predictions about each informant’s vote and belief before asking them whether the consensus was accurate, as we did; instead, it just stipulated a unanimous sequence of votes, and asked whether participants’ inferences fit a model in which their informants’ judgments were Bayes-normative.

With that caveat in mind, we still think our computational model sheds light on past work, for two reasons. First, it can represent the kind of Bayes-normative informants that participants have been compared with in past work, by setting *ω*_acc_ = 1.0 (which nullifies the *θ*_socCon_ and W_Power_ terms, meaning informants always vote their beliefs) and setting W_Self_ = 1.0 (so that no single informant treats their initial belief as stronger evidence than any other informant’s vote). This is particularly notable because the region a Bayes-normative sequence would show up in (the top right quadrant in Figure 3c) is almost completely empty in our experiments. In other words, it’s probably not safe to assume that participants in psychology experiments expect their informants to make Bayes-normative judgments. Second, our model suggested that participants in our experiments generally expected their informants’ initial beliefs to carry enough weight that a short sequence wouldn’t be able to generate a vote-flipping cascade unless an early speaker commanded quite a bit of deference. And short of accurately computing the conditional probabilities a Bayes-normative informant would get from early speakers’ votes, participants in past work wouldn’t have any reason to suspect that kind of deference.

We take this to suggest that one reason for the mixed evidence on whether people make Bayes-normative inferences about sequential consensus is that they generally don’t expect their informants’ public judgments to misrepresent their private beliefs *without strong cause*—and they are particularly vigilant about *ir*rational causes (e.g., social favor). To the extent that people’s vigilance is calibrated to the kinds of causes that make sequential testimony *less* reliable in the real world (e.g., motivational biases, Mercier & Milton, 2019; Oktar et al., 2024), they may default to assuming the absence of such causes in experimental procedures that don’t include evidence of them. The bottom line: a model of sequential consensus that assumes Bayes-normative informants may thereby misrepresent the learning problem people face in the real world, and underestimate people’s sensitivity to unreliable sequences. Our results suggest that non-epistemic influences like social favor may make people more vigilant.

One assumption built into our computational model and study design is worth flagging as both a potential limitation and direction for future work: the model allowed the best-fitting parameters to vary by participant, but not by informant (with the exception of W_Power_ in the varPower model, which could vary for the Popular informant). That simplifies model fitting, but it also flattens some of the richest intuitions people have about why their informants agree or disagree (e.g., competence, character, relationships) into a one-dimensional “typical” informant (Danovitch & Keil, 2007; Harris et al., 2018). And there’s reason to believe that it’s those rich intuitions about how different voters might react to different sequences of votes that really bring people’s inner Machiavelli to life (Davis et al., 2022, 2023; Pietraszewski, 2021; Thomas et al., 2022).

To illustrate: suppose a 5-person steering committee’s only pre-sequence Yellow vote is Donny T, a powerful party leader. The four Blue votes come from Lindsey G, who you think is a lickspittle (i.e., *ω*_acc_ = 0.0), and the three other members, who have always loudly claimed to put truth before party (i.e., *ω*_acc_ = 1.0 for all three). As party whip, you control speaking order. What can that do for you? If three voters are truly at *ω*_acc_ = 1.0, no speaking order can overturn the Blue majority. But, you’ll guarantee Donny two votes if you allow he and Lindsey to speak first and second. You can’t be as sure of Lindsey’s vote if you put him last, but watching him decide whose boots to lick would tell you whether Donny has enough leverage over him to make him vote against his beliefs even when he already knows he’d have the other three members on his side. Finally, putting Donny last guarantees Blue 4 votes, but it also tells you whether he’s willing to try to face down his whole party. We found some evidence of that final scenario in Experiment 3: participants were more likely to expect the dissenter to vote his beliefs when he was Popular than Anonymous—even though in our model, the dissenter’s W_Power_ only affects *other* informants’ votes, not his own. Without by-informant parameters (e.g., for W_Self_), what looks like a commonsense inference to human intuition is just adding noise to the model. Future modeling work could examine those inferences more closely by fitting by-informant parameters during a training phase; they could also be examined experimentally by manipulating the first half of a sequence and either asking participants to *predict* the second half or *infer the causes* of a given second half—in other words, can laypeople identify the “pivotal vote” in a sequence from speaking order?

Where does this leave us? Go back to our restaurant example: to the extent that Alice’s real world experience makes her discerning enough to know when Bob’s vote is too strongly anchored to his initial beliefs for Carol’s vote to flip it, it also justifies her in thinking that the evidence she gets from seeing them agree that Louis’ Lunch makes a better burger isn’t worth any less just because they voted in sequence—after all, they *would have* said the same thing independently. On the other hand, evidence that people are able to predict how speaking order and social favor can change people’s public and private judgments points to a different class of worries: strategic voting, agenda manipulation, and information gerrymandering (Arrow, 2012; Levine & Plott, 1977; Stewart et al., 2019). In any democratic procedure for choosing between more than two options, people may end up voting for the option they think they can get (or against one they don’t like) instead of the option they most strongly prefer—allowing a “spoiler” to beat the option most voters prefer (Arrow, 2012). Our work suggests that even in two-option decisions, social favor may become a third wheel. So while knowing when her informants’ votes are motivated by social favor more than accuracy may help Alice detect pluralistic ignorance, it can also make her especially effective at manipulating outcomes if she’s given the chance to set the agenda—like the party whip who can choose who votes when or which options get voted on first, thereby forcing people to vote strategically because of uncertainty about how many votes they have on their side (Plott & Levine, 1977; Stewart et al., 2019). But those Machiavellian intuitions don’t make Alice’s manipulations invisible: other voters may be just as capable as she is of recognizing when public consensus misrepresents private beliefs. And while research on sequential reasoning treats belief updating as a one-way street, real-world contexts provide plenty of opportunity for people to share second thoughts in back-and-forth discussion—making pluralistic ignorance a challenge to maintain in deliberative groups (Mercier & Claidière, 2022; Richardson & Keil, 2022; Trouche et al., 2014). We suggest that research on laypeople’s trust in non-independent consensus (Whalen et al., 2018; Xie & Hayes, 2022; Yousif et al., 2019) might benefit from considering that trust in light of their reasoning about when and why public and private judgments diverge.

## Acknowledgments

This work was supported by funding to Frank Keil from Yale University. 

## Data Availability Statement

The data are available on the first author’s OSF repository (https://osf.io/fnz2s/overview), along with data, materials, and R scripts for analysis & visuals.

## Note

^1^ Note that, by using a 1-20 scale with discrete increments of 1, participants were not able to rate an informant as equally likely to vote either way, which would correspond to a rating of 10.5* Pre-registrations are available on the first author's OSF repository (https://osf.io/fnz2s/overview?view_only=35b38cd36b524545bb666a729a52ce56) along with the data, analyses, and all other materials.

## Supplementary Material


