# Supplementary material for: Agenda Setting and The Emperor’s New Clothes: People Diagnose Information Cascades During Sequential Testimony by Reasoning About Informants’ Speaking Order and Social Status
Source: Open Mind (Camb). 2025 Nov 22;9:2005–30. doi: 10.1162/OPMI.a.258 (PMC12768551; doi:10.1162/OPMI.a.258)
Supplement: Supplementary file 1 [file opmi-09-2005-s001.docx]

**SI.1-2: Simulation Results**

The model’s predictions for each speaker are constrained on two sides: first, by the parameters themselves, and second, by the pattern of previous speakers’ votes. However, some combinations of parameters make some vote patterns unlikely or impossible: for instance, when parameter values are low for both accuracy and self weight (ω_Self_ > ω_Power_ or ω_acc_socFav_ <= 0.5, respectively), the model only expects Spkr3 to vote Blue if Spkr2 did as well — but under those parameter values, Spkr2’s probability of doing so approaches null. In order to understand the model’s dynamics even in low-probability vote patterns, we simulated each speaker’s predicted vote independently at each round (e.g., even if a given parameter set made the probability of a Y vote from Spkr2 negligible, we still simulated Spkr3’s vote under both a YY and a YB pattern).

Figure SI.1 below shows the full set of vote patterns when the private dissenter speaks first. As in the main text, we considered four distinct regions of the parameter space in which the model makes qualitatively different predictions. These four regions are defined by whether or not participants expect accuracy to outweigh social favor or vice versa (**ω_acc_socFav_** > 0.50), and whether or not they expect the informants’ own initial judgment to outweigh the first speaker’s vote (**ω_Self_** > **ω_Power_**). As can be seen from the proportion of estimates above the midpoint of the y-axis in the figure, speaking first often allows the private dissenter overturn the initial consensus entirely, especially when speakers don’t value accuracy more than social favor (i.e., **ω_acc_socFav_** <= .50), or when the first speaker’s popularity outweighs the subsequent speakers’ trust in their own initial judgment (**ω_Self_** <= **ω_Power_**). The simulation also suggested that allowing the dissenter to speak first makes Spkr2’s vote pivotal: a Yellow vote from Spkr2 nearly guarantees a unanimous consensus regardless of the other parameter values, while a Blue vote from Spkr2 frequently keeps any other subsequent speaker from voting Yellow either unless social favor outweighs accuracy (**ω_acc_socFav_** <= 0.50) or the dissenter’s power outweighs the informants’ initial judgment (**ω_Self_** <= **ω_Power_**).


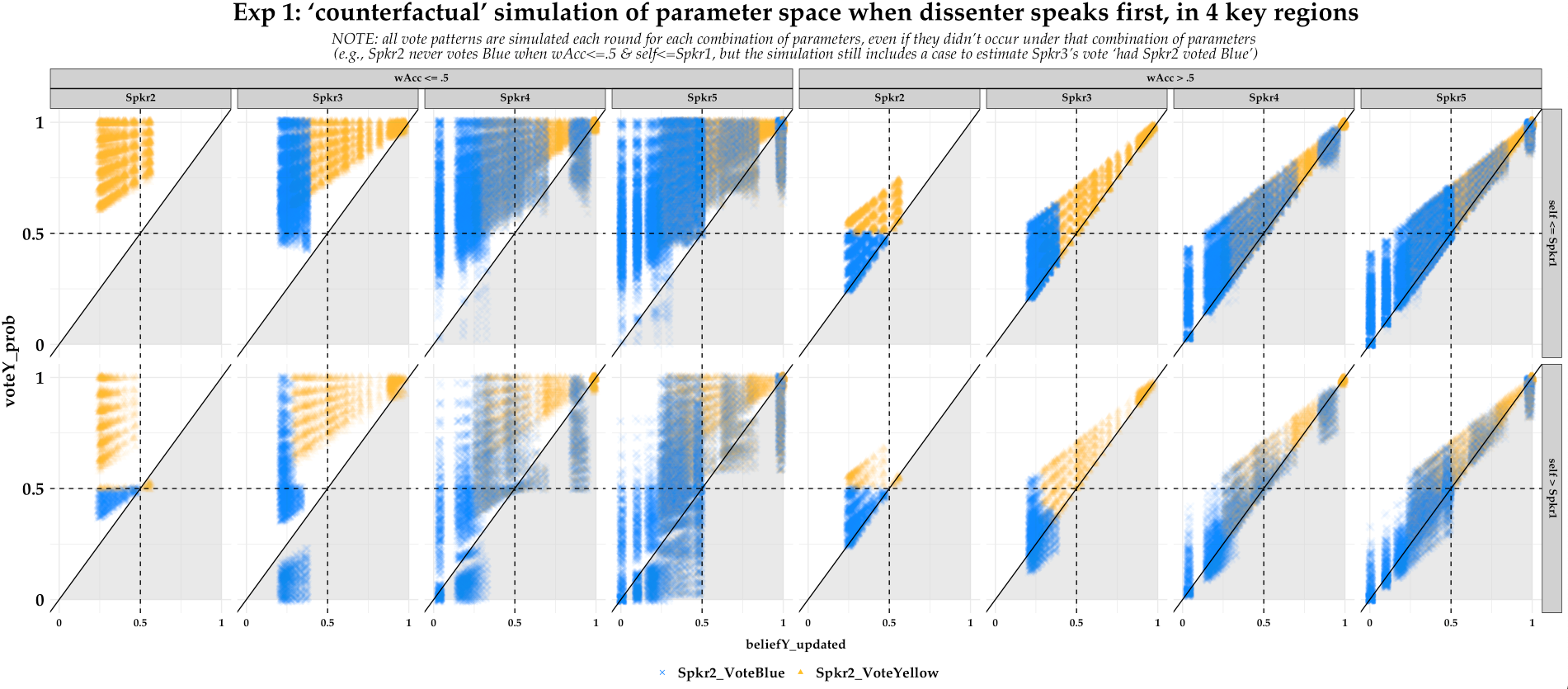


**SI.1: Simulation Visualization (Exp 1)**

**Figure SI.1.1**: Simulation of all parameter combinations and vote patterns, including ‘counterfactual’ patterns (e.g., both YY and YB for Spkr3 even for parameter sets in which Spkr2 never votes Y). Predictions about Spkr2’s vote are propagated through the remainder of the sequence in qualitatively different ways for different regions of the parameter space defined by whether or not accuracy outweighs social favor (wAcc > .5 versus <=. 5) and whether or not informants’ own beliefs outweigh Spkr1’s vote (wSelf > Spkr1 versus wSelf <= Spkr1). The diagonals divide predictions that favor Yellow more strongly publicly than they do privately (upper, unshaded), or vice versa (lower, shaded).

Figure SI.2 shows the full set of vote patterns when the private dissenter speaks last. As can be seen from the identical distributions of responses when ω_Self_ > ω_Power_ and vice-versa, speaking last prevents the dissenter’s popularity from having any effect at all on previous speakers. According to the model, Spkr2 is far more likely to vote Blue than Yellow regardless of parameter combination; and even when we force the counterfactual in which Spkr2 did vote Yellow, Spkr3 is still more likely to vote Blue than Yellow under almost all parameter combinations. Notably, the model’s confidence that speaking last would lead the dissenter to both publicly and privately endorse the consensus contradicts participants’ judgments, who almost unanimously expected Spkr5 to privately dissent, and sometimes expected him to publicly dissent as well — especially if he was popular.


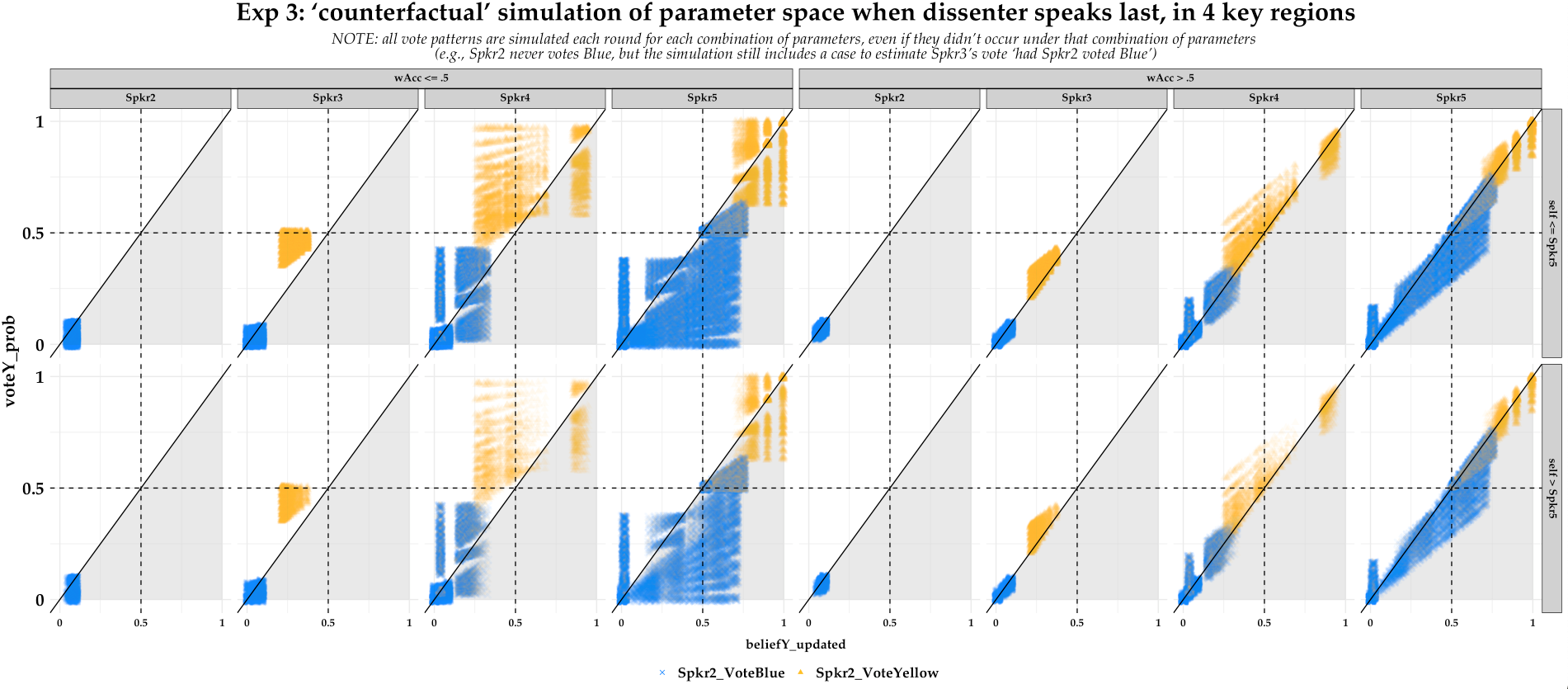


**SI.1: Simulation Visualization (Exp 3)**

**Figure SI.2**: Simulation of all parameter combinations and vote patterns, including ‘counterfactual’ patterns (e.g., both YY and YB for Spkr3 even for parameter sets in which Spkr2 never votes Y). As in Figure SI.1, facets are defined by whether or not accuracy outweighs social favor and whether or not the speakers’ own beliefs outweigh private dissenter’s vote. The diagonals divide predictions that favor Yellow more strongly publicly than they do privately (upper, unshaded), or vice versa (lower, shaded). Since the private dissenter votes last, the four key regions no longer differ in how Spkr2’s vote propagates through the remainder of the sequence.


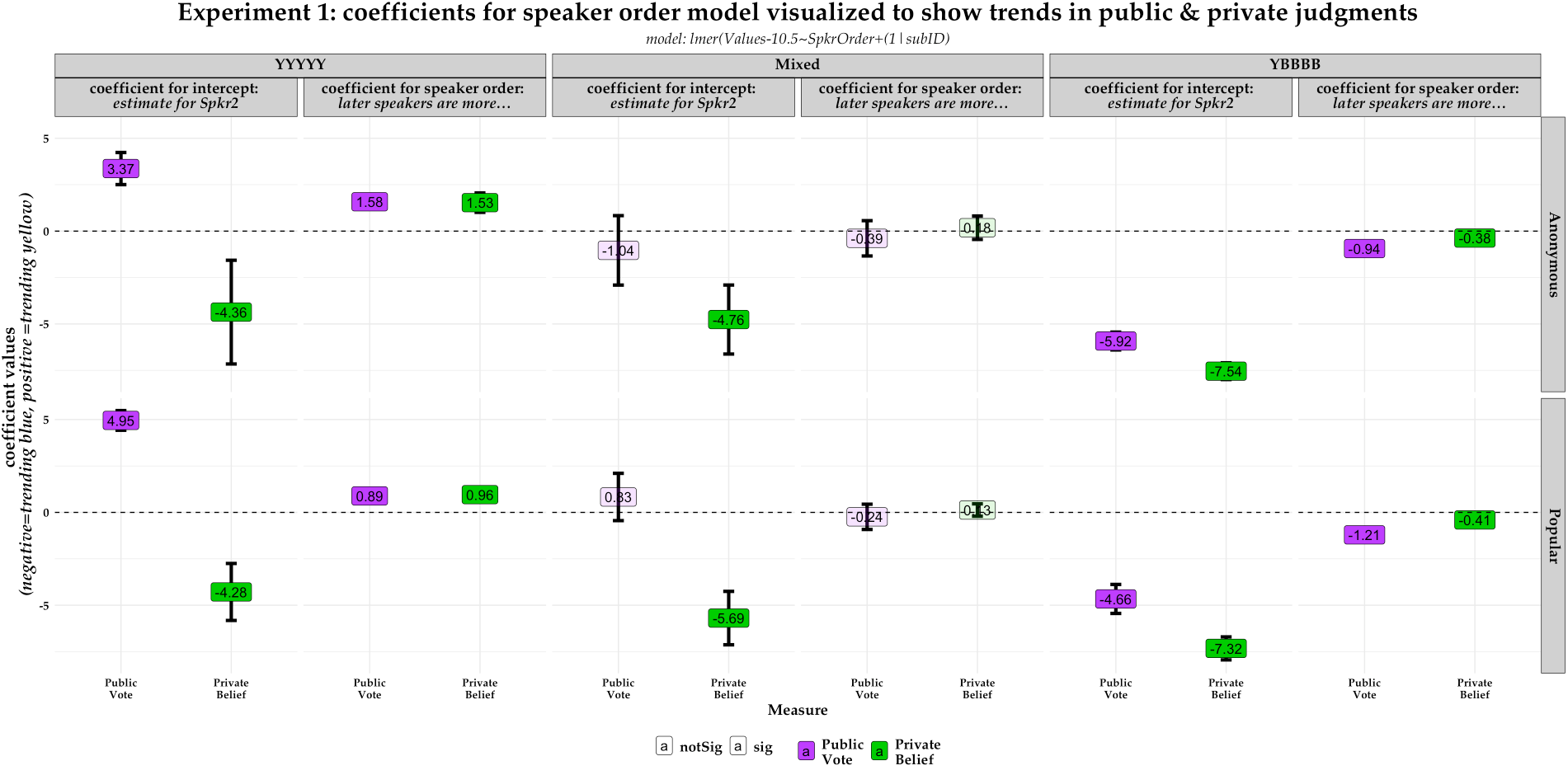


**SI.3: Visualization of Exp 1 analysis in manuscript**

**Figure SI.3**: Visualization of the model discussed in the analysis of Experiment in the manuscript (ratings_centered~speaker_order, with random intercepts by participant). The regression was run separately for each vote pattern (YYYYY, Mixed, YBBBB), status (Popular, Anonymous), and judgment type (Public, Private). The y-axis shows the estimated coefficient values (positive = endorsing Spkr1’s Yellow vote, negative = contradicting Spkr1’s Yello vote), and the coefficients for are mapped to the subheadings (intercept = estimates for Spkr2, speaker order = trend for later speakers relative to Spkr2). Regardless of whether Spkr1 is Popular or Anonymous, participants expect Spkr2 to *privately* endorse Blue (intercept, green). But participants who expected Spkrs2-5 to *all* publicly endorse the same option (YYYYY or YBBBB) also expected the later speakers’ public and private judgments to shift towards whichever option Spkr2 *publicly* endorsed, while participants who didn’t expect Spkr2-5 to all make the same public judgment also didn’t expect later speakers to shift in one direction instead of the other.


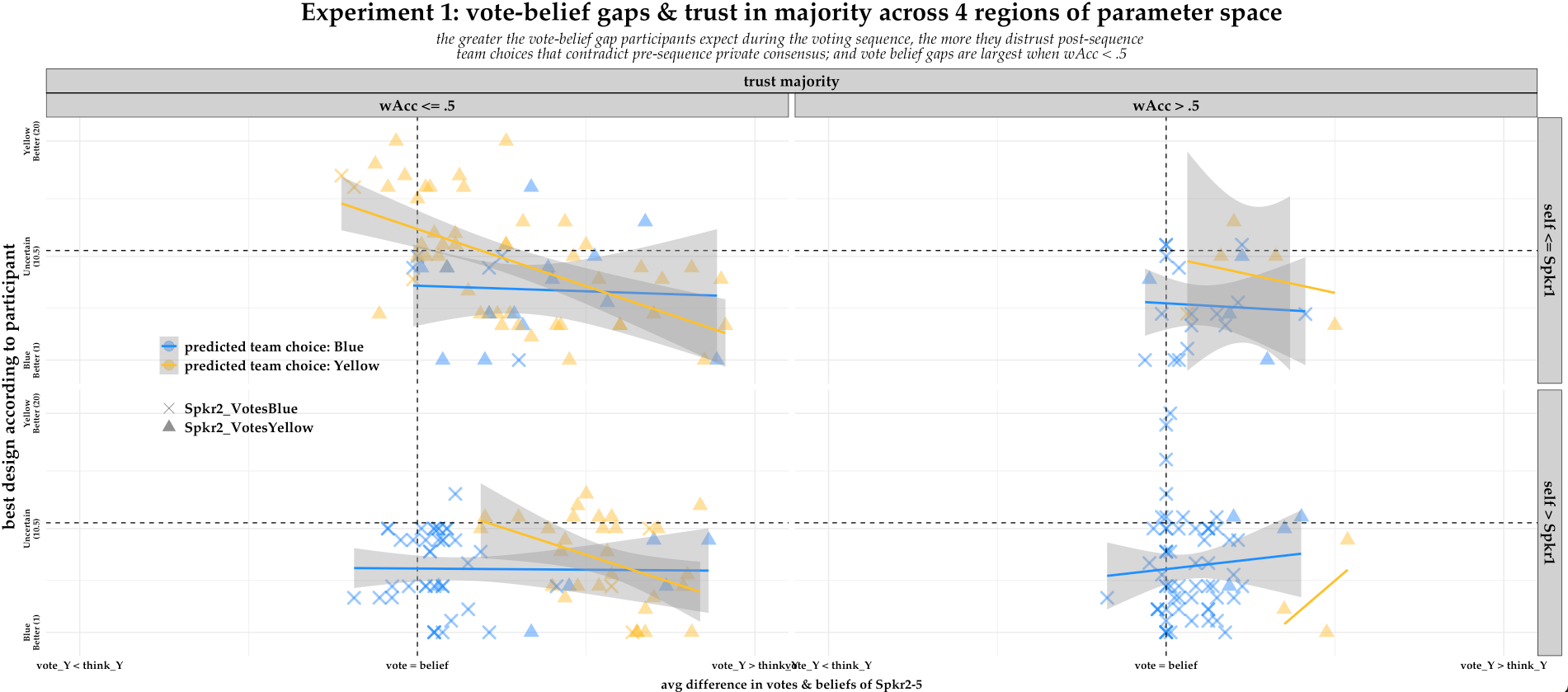


**SI.4: Visualization of Exp 1**

**Fig SI.4.** The relationship between the design the participant endorsed (y-axis), the gap they predicted on average between the informants’ votes and beliefs duing the sequence (x-axis), and the participants’ prediction for Spkr2’s vote (shape), with separate linear fits for the team’s predicted final decision after the post-sequence discussion (color). The facets separate participants into 4 regions of the parameter space whether W_Power_ < W_Self_ or vice-versa, and whether ω_acc_ <= 0.5 or > 0.5): nearly all participants who expect the final team choice to contradict the pre-sequence consensus have low ω_acc_ values, and among those participants, trust in the final team choice decreases as the vote-belief gap they expected during the sequence increases.


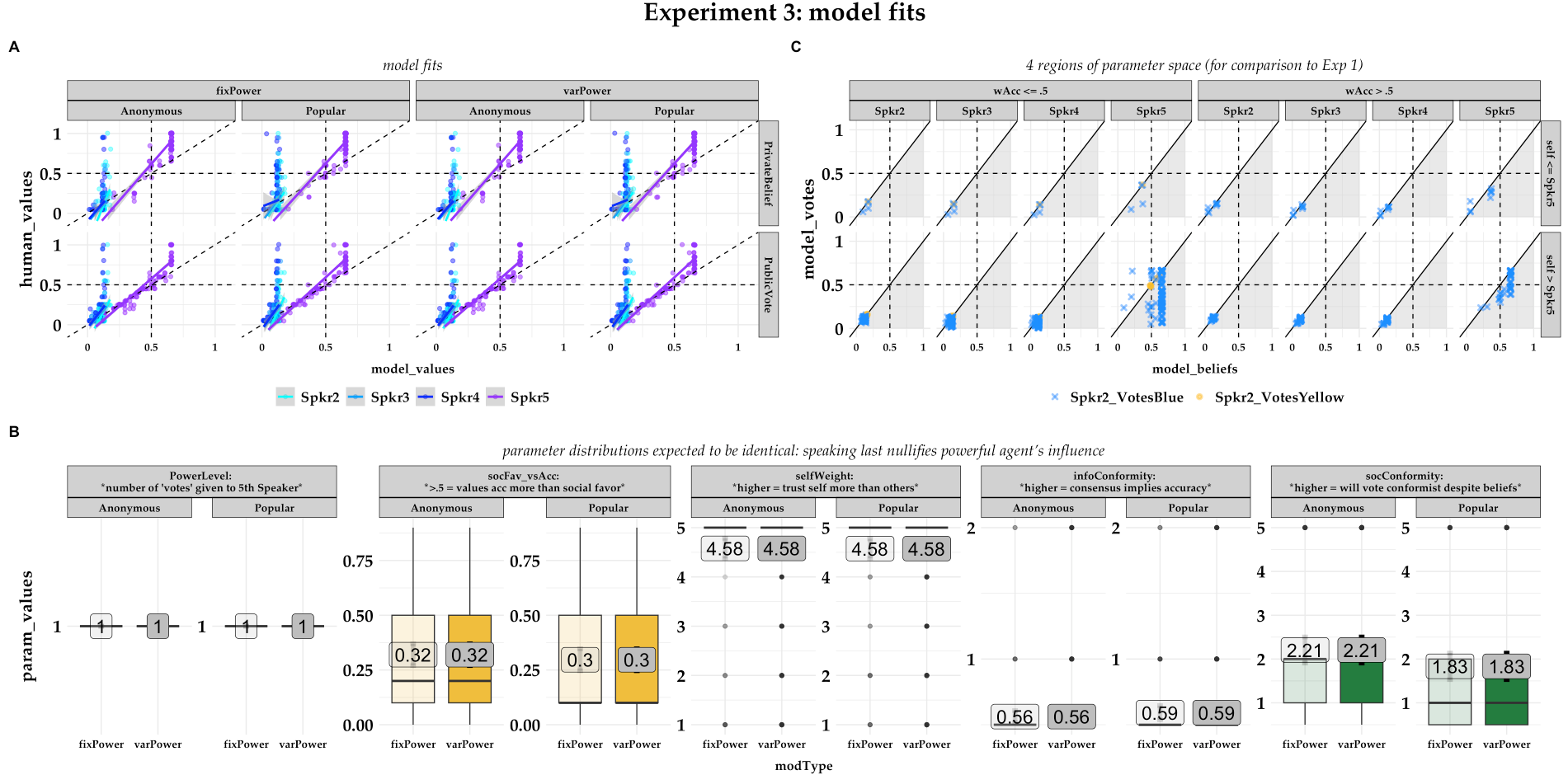


**SI.5: Model Fits for Exp 3**

**Figure SI.5**: **Panel A**: Model fits for participants’ inferences about each speaker’s public and private judgments in each condition of Experiment 3. **Panel** **B:** Distribution of optimal fitting parameters for participants in each condition of Experiment 3 according to the fixedPower (faded) and varPower model (dark), as reported in main text. As expected, distributions are identical, because the model assumes the popular informant can only influence subsequent speakers — speaking last nullifies their influence. **Panel C**. For comparison with Exp 1, where the varPower model’s predictions about Spkr2’s vote are propagated through the remainder of the sequence in qualitatively different ways for different regions of the parameter space defined by whether or not accuracy outweighs social favor (wAcc > .5 versus <=. 5) and whether or not informants’ own beliefs outweigh Spkr1’s vote (wSelf > Spkr1 versus wSelf <= Spkr1). The diagonals divide predictions that favor Yellow more strongly publicly than they do privately (upper, unshaded), or vice versa (lower, shaded). In Experiment 3, informants overwhelmingly endorse Blue publicly and privately.
